# Coping with Emotional Distress via Self-Disclosure to Robots: An Intervention with Caregivers

**DOI:** 10.1007/s12369-024-01207-0

**Published:** 2025-05-26

**Authors:** Guy Laban, Val Morrison, Arvid Kappas, Emily S. Cross

**Affiliations:** 1 Department of Computer Science and Technology, University of Cambridge, Cambridge, UK; 2 School of Psychology and Neuroscience, University of Glasgow, Glasgow, UK; 3 Department of Psychology, Bangor University, Bangor, UK; 4 School of Business, Social and Decision Sciences, Constructor University, Bremen, Germany; 5 MARCS Institute for Brain, Behaviour and Development, Western Sydney University, Sydney, New South Wales Australia; 6 Professorship for Social Brain Sciences, ETH Zürich, Zürich, Switzerland

**Keywords:** Social robots, Human-robot interaction, Caregiving, Longitudinal, Self-disclosure, Informal care, Emotion regulation, Distress

## Abstract

People often engage in self-disclosure and social sharing when trying to cope with emotional distress. This study introduces a novel long-term intervention designed to help informal caregivers cope with emotional distress by self-disclosing towards a social robot. Research indicates that informal caregivers frequently face challenges in handling the emotional and practical demands of caregiving, often experiencing a lack of social support and limited social interaction. Accordingly, we explored the extent of informal caregivers’ self-disclosure behaviour towards a social robot (Pepper, SoftBank Robotics) over time, and how their perceptions of the robot evolved. Additionally, we examined how this intervention affected caregivers’ moods, perceptions of the robot as comforting, feelings of loneliness, stress levels, as well as its impact on their emotion regulation. We replicated a previous long-term experiment [1] with a dedicated sample of informal caregivers who interacted with Pepper 10 times over five weeks, discussing everyday topics. Our results show that caregivers increasingly self-disclosed to the robot over time, perceiving it as more social and competent. Participants’ moods improved following interactions, and they viewed the robot as increasingly comforting. They also reported feeling progressively less lonely and stressed. Thus, our findings with informal caregivers replicated those of [1]. Moreover, after the intervention, caregivers reported greater acceptance of their caregiving roles, reappraising it more positively, and reduced feelings of blame towards others. These results highlight the potential of social robots to provide emotional support for individuals coping with emotional distress.

## Introduction

Emotional distress can be defined as an unpleasant emotional state that occurs when one has limited abilities or is unable to adapt to stressors and to their consequences, both perceived and actual [2]. As a wealth of literature attests, emotional distress is one of the most common human emotional experiences. It can arise from various situations and stressors ranging from unexpected calamities (e.g., grief and loss, natural or man-made disasters, or physical or mental illness) to typical annoying daily events [3]. The feeling of persistent emotional distress widely and negatively impacts people’s well-being, and carries other mental and physical health implications [4] ranging from psychiatric disorders and psychopathologies (e.g., depression and anxiety) [5] to immune system dysfunction [6]. One meaningful way of coping with emotional distress and its associated comorbidities is by exercising emotion regulation, a set of internal and external processes and techniques that involve monitoring, assessing, and modifying one’s state behaviour or cognition in a given situation [7]. While some self-regulation techniques (e.g., reappraisal, affect labeling, etc.) are highly supportive, during difficult times people are often prone to adopting maladaptive emotion regulation strategies (e.g., suppression) [8] that can have long-lasting negative effects on their well-being [9]. For many, exercising constructive emotion regulation is not an easy task as it can be resource-demanding [10–12] and engaging in various forms of interpersonal communication behaviours like self-disclosure and social sharing with others effectively support this process [13–15]. Self-disclosure is a communication behaviour aimed at introducing and revealing oneself to others, and it plays a key role in building relationships between individuals [16, 17]. Numerous health advantages have been associated with engaging in different types of self-disclosure, including the ability to elicit and provide support and improve mood, and offer a comfortable setting for sharing feelings [13–15, 18, 19].

One group of people that has been shown to be particularly prone to emotional distress is informal caregivers [20–23]. Informal caregivers provide care and support to a friend or family member while typically being unpaid and non-formally trained. Their care recipients often suffer from chronic health conditions that are related to old age or a variety of physical and mental health conditions [24]. While many informal caregivers find the caregiving experience to be rewarding [25, 26], this experience is also often associated with serious health and well-being implications for the informal caregivers themselves [24, 27, 28]. The caregiving situation is considered to be a potential stressor [20], which might lead to a variety of negative health and well-being outcomes including physical and emotional strain, burden [related to the caregiving task; see meta analysis and review [29, 30]], and depression [31, 32]. The role of a caregiver, which requires time and resources [20, 24, 33], can limit informal caregivers from receiving professional mental and physical health support for themselves. This is a substantial psychological factor as caregivers struggle with managing stress and practical demands of the caregiving role while experiencing loss (of a person in terms of the care recipient *“former self"*, or of their independence) and not receiving the necessary help [23, 28, 34]. Above all, due to the loss of a healthy and independent significant other, increased care and family responsibilities, shrinking personal space and reduced social engagement, informal caregivers often report experiencing a tremendous sense of loneliness, which can further impact their ability to self-disclose their emotions and needs to others [35–38].

Accordingly, in this study, we replicated a long-term experiment involving a social robot [1], modifying it to serve as an intervention for a group of informal caregivers. We used the same behavioural paradigm and experimental setup of [1], with several modifications (see Sect. 3.1). Our goal was to investigate how engaging informal caregivers in self-disclosure through regular conversations with a social robot could help them cope with emotional distress over time. Social robots are autonomous or semi-autonomous machines that interact and communicate with humans or other agents by following social behaviours and rules relevant to their role [39]. These robotic agents can take on various forms and shapes and are gradually being deployed across various health and well-being settings due to their ability to function autonomously or semi-autonomously in physical and social spaces alongside humans [see [40]]. Social robots are being increasingly studied in psychosocial health interventions [see [41]], including those oriented for supporting mental health [see [42, 43]], rehabilitation [44], and providing much-needed physical and social support across a number of daily life settings [40]. Due to social robots’ social features [45, 46], animate qualities [47, 48] and physical and social embodiment [49], previous studies provide evidence for how social robots might be useful for encouraging humans to self-disclose information and emotions [e.g., see [1, 50, 51]] and provide a sense of companionship to individuals (such as informal caregivers) who could use the support of socially-savvy artificial agents [52].

Given the importance of self-disclosure for psychological health and the potential benefits it offers to informal carers dealing with emotional distress, we aim to replicate the methodology and results of [1] with a narrower and dedicated population of informal caregivers. Accordingly, we are asking:

*RQ: To what extent does self-disclosing to a social robot across several sessions over the course of 5 weeks impact informal caregivers’ self-disclosure behaviour toward the robot, perceptions of the robot, and their emotional well being and emotion regulation tendencies?*

To answer this research question, we replicated [1], conducting a mediated long-term online experiment with informal caregivers conversing with a social robot 10 times over 5 weeks about general everyday topics. We used several objective and subjective measures to evaluate the extent of self-disclosure towards the robot, how it was perceived over time, and how it affected informal caregivers’ emotional well-being and emotion regulation. Beyond addressing the aforementioned objectives, we chose to replicate [1] because replicating Human–Robot Interaction (HRI) studies is essential for confirming the validity and generalisability of behavioural and social findings. HRI replication studies ensure that robots are well-suited for their intended social roles [53, 54], particularly when employed in interventions [40].

## Theoretical Framework and Related Works

The map of interpersonal regulation [15] explains that people might use self-disclosure as an intrinsic regulatory process to achieve various goals, which can be response-dependent or independent. In intrinsic response-dependent regulation, one might self-disclose to a conversation partner to seek feedback that supports their regulatory attempt, such as an empathetic response or confirmation. Previous research highlights that seeking support and concealment via disclosure can positively affect people’s mood and help them cope with emotional events [e.g., [55, 56]]. When engaging in intrinsic response-independent regulation via self-disclosure, individuals seek a channel for disclosure regardless of potential responses or feedback. Accordingly, the mere act of disclosure contains certain psychological components that affect regulatory success. Previous research highlights the importance of feeling listened to and how it can impact various aspects of well-being, such as feelings of loneliness [57] and perceptions of burden [58]. When sharing with others solely for the sake of disclosing emotions and feelings, individuals might engage in appraising their own emotions and experiences, thereby dampening the intensity of the emotional experience [15].

This strategy is also known as affect labeling, a simple and implicit emotional regulation technique aimed at explicitly expressing emotions, or in other words, putting feelings into words [59, 60]. Accordingly, people use self-disclosure for emotional introspective processes, self-reflecting on their emotional experiences as well as past behaviours and actions [61]. A similar example is James Pennebaker’s writing disclosure paradigm [see [62, 63]], which helps people regulate their emotions by writing about their own experiences. These types of self-disclosure behaviours are found to be highly useful for coping with emotional distress [59–61, 64–67].

### Self-Disclosure as an Intervention for Informal Caregivers

Due to the emotional distress caregivers experience, they often engage in suppressive behaviours like *protective buffering* [68–70] hiding their worries and denying their concerns [71] aiming to protect their care recipients from negative information and keeping it to themselves [72, 73]. This maladaptive emotion regulation strategy [suppression; [74]] is associated with negative social [75], psychological and health-related outcomes [9], and can drastically impact symptoms of depression and anxiety [76]. Accordingly, few studies have tested interventions for informal caregivers using intrinsic interpersonal emotion regulation techniques to promote self-disclosure and reduce suppression and other maladaptive behaviours. One study with two randomized controlled trials with informal caregivers (N = 38) going through six structured telephone-based support group meetings showed promising results with regard to caregivers’ self-rated psychological Health-related quality of life [77]. Another observational study reported that the presence of friends and social interactions with other people (that are not within the dyad) supports carers’ well-being [78]. A systematic review by Dam and colleagues [79] explains that there is limited evidence for social support interventions for informal caregivers (via peer support, family support and social network interventions, support groups and remote interventions using the internet or telephone). Nevertheless, a nine-year panel survey reveals that participating in social activities with others can effectively reduce informal caregivers’ emotional distress [80]. Notably, growing evidence shows that online support groups and social media channels can help informal caregivers cope with emotional distress by practising intrinsic regulation techniques and self-disclosing their experiences with the caregiving community [e.g., [81–84]].

### Social Robots for Emotional Support

While numerous approaches for supporting and implementing emotion regulation training have been proposed and trialled [see [85]], considerable economic, logistical [see [86, 87]], professional [see [88]], and socio-emotional barriers [see [89]] can limit individuals from receiving appropriate treatment. In recent years, numerous emerging technologies and digital interventions have been studied and tested, showing unique opportunities to support emotion regulation in various contexts [90, 91].

While some digital interventions for informal caregivers have been introduced in the past [see [92]], interventions facilitated via richer modalities of communication, such as flowing dialogue [see [93, 94]], can impact users’ perceptions of the system and provide a better user experience than non-interactive systems [e.g., [95]]. Social robots build a sense of rapport [96] by displaying (verbal and nonverbal) social cues [e.g., [50]], while preserving a sense of anonymity and confidentiality [97] which creates a safe and comfortable environment for disclosing emotions [e.g., [98, 99]; see [51]]. Therefore, social robots might just fall at the ideal intersection between being an autonomous or semi-autonomous and physically present technology [see [40]] that can capture emotion [100, 101] while also being able to demonstrate social and cognitive cues that might help to respond correctly to those who suffer from emotional distress [43]. Accordingly, many studies show the benefits of employing social robots as alternative self-managed interventions for providing emotional support [see reviews, [100–102]].

For example, in a recent study, a social robot was employed to mediate a single-session loving-kindness meditation and walking meditation, oriented to counter symptoms of depression among young people. The study reports that the interventions were successful in evoking state openness, with the former also exerting a positive effect on valence [103]. Several other studies have been addressing long-term interventions for people’s well-being, reporting on how these interactive agents might support people in different ways. Bodala and colleagues [104] employed a social robot delivering teleoperated mindfulness coaching for five weeks. An additional example includes Axelsson and colleagues [105] that tested a robotic coach conducting positive psychology exercises, showing positive mood change after participation in the intervention. Another pilot randomized controlled trial found that a humanoid social robot delivering a brief mindful breathing technique demonstrated initial feasibility and high acceptability among young adults [106]. Evidence shows that social robotic interventions can successfully induce both behavioural and cognitive changes. In a series of studies, Robinson and colleagues used social robots to deliver behaviour change interventions, employing verbal motivational techniques to reduce high-calorie snack consumption. The studies demonstrated promising results, evidenced by objective measurements like weight loss [107], and qualitative insights highlighting the subjective experiences of participants [108]. These interventions also showed promising outcomes in helping patients with diabetes manage their own care over an 8-week period [109].

Studies applying robotic interventions for supporting people’s emotional distress are rarely taking place in people’s homes, and are often conducted in laboratories. One successful example of a field experiment is a study employing the social robot Jibo as a positive psychology coach to improve students’ psychological well-being in students’ on-campus housing. The study results describe a positive effect on students’ well-being with positive mood change, and also students expressing their motivation to change their well-being [110]. Another study employed two robotic coaches of different embodiment (QTrobot with a human/child-like embodiment, and Misty robot with a more machine-like embodiment) for promoting mental well-being in organizational settings. The results of the study indicate that participants perceived the robot Misty more positively than QTrobot and felt a stronger connection with it [111].

We conducted a mediated long-term online experiment where participants conversed with a social robot 10 times over 5 weeks. Our findings indicated that people increasingly self-disclosed to the social robot over time and perceived the robot as more social and competent. Participants’ moods improved after talking to the robot, and across sessions, they found the robot’s responses more comforting. Additionally, they reported feeling less lonely over time [1]. These results highlight the potential of social robots to engage in intrinsic interpersonal emotion regulation by encouraging self-disclosure. Another notable example includes two studies that found people in a bad mood benefited more from disclosing to a robot than from writing disclosures in a journal [112] or on social media [113]. These findings correspond with other empirical results in the field, suggesting that people who experience negative emotions tend to self-disclose more towards social robots [114]. When comparing to disclosures to humans, we previously found that people shared more information with a human than with a humanoid social robot [50]. Yet, a different study by [98] found that interactions with a social robot elicited lower tension compared to interactions with a human. The same study [98] showed the benefits of employing social robots for minimising social tension and anxieties, describing that participants with higher social anxiety felt less anxious and demonstrated less tension when knowing that they would interact with a robot as opposed to a human interlocutor. Another study by Barfield [115] investigated whether robots that self-disclosed personal information encouraged reciprocal self-disclosure from users, emphasising the importance of trust, context, and anthropomorphism in shaping these interactions.

### The Current Study

Due to their caregiving responsibilities, informal caregivers rarely have a non-judgmental space to conveniently disclose their emotions and needs [23]. We aimed to address this need using an empathetic social robot, even if the robot was lacking in feedback [see [15], Response-Independent Processes]. Thus, in this study, we aimed to replicate Laban et al. [1] by using the behavioural paradigm from the original study as an intervention. Our goal was to elicit rich self-disclosures from informal caregivers through repeated verbal interactions with a social robot. Following [1] results, we hoped this would give caregivers the chance to reflect on their stressors and worries, use the robots as a listening ear, and provide a convenient channel for disclosure. This, in turn, would help them regulate their emotions more effectively. Beyond replicating the results of [1], we sought to demonstrate the potential of using a social robot in interventions to support intrinsic interpersonal emotion regulation.

Following [90] recommendations, we aspired to leverage social interaction with the robot to promote social-affective communication. Instead of using social robots merely as companions [e.g., [52]], we employed a social robot to encourage and listen to people’s self-disclosures. By engaging informal caregivers in self-disclosures to a social robot-talking about themselves and their lives-we expected them to engage in intrinsic interpersonal emotion regulation [e.g., via affect labelling, [59]], avoid suppressive behaviours [see [74]], and reflect on their lives and caregiving experiences.

We previously reported the development of the paradigm, the experimental design and procedure, and empirical results from a non-caregiver sample [1]. A summary of these results can be found in Sect. 2.2. Following recommendations and guidelines for rigorous HRI research and science [40, 45, 54, 116], we replicated the study [1] with an informal caregiver sample. This was done to further validate our previous findings and assess the suitability of this paradigm as a potential intervention to support emotional well-being among a population highly prone to emotional distress [20]. Further details on the changes and adaptations between this study and the replicated study can be found in Sect. 3.1. Our objectives were to test the following aspects and achieve the following goals:

- **(O1)** Due to the negative health outcomes associated with suppressive coping behaviours [9] and the tendency of informal caregivers to engage in these behaviours [see [68, 71–73]], we aim to study how repeated interactions with a social robot influence informal caregivers’ self-disclosure behaviour toward the robot. Based on our previous findings [1], we expect that informal caregivers will gradually open up and increasingly self-disclose to the robot over time.
- **(O2)** To evaluate the feasibility, adaptation, engagement, and potential for building relationships through long-term interactions with social robots as interventions for coping with emotional distress, we will assess informal caregivers’ social perceptions of the robot and their user experience. Based on our previous findings [1], we expect that caregivers will gradually perceive the robot as more social and competent over time.
- **(O3)** To better understand the effectiveness of the intervention and how self-disclosure to social robots can improve informal caregivers’ well-being and reduce emotional distress over time, we will evaluate the impact of repeated interactions with the social robot on emotional well-being. Consistent with [1], we expect the intervention to positively affect informal caregivers’ well-being.
- **(O4)** Finally, we will assess the intervention’s effect on informal caregivers’ cognitive changes in emotion regulation. Specifically, we aim to examine the extent to which informal caregivers adapt and change their emotion regulation skills, strategies, and thoughts due to their participation in the intervention.

## Methods

Consistent with previous proposals [117, 118], we pre-registered the study and report for how we determined our sample size, all data exclusions, all manipulations and all measures in the study [see [119]]. In addition, following open science initiatives [e.g., [120]], the de-identified data set, stimuli and analysis code associated with this study are freely available online [see [121]]. By making the data available, we enable and encourage others to pursue tests of alternative hypotheses, as well as more exploratory analyses.

Since this study replicates the behavioural paradigm and experimental procedure of [1], but with a different participant population, the following methodology section gives a brief summary of these methods, with a particular focus on any differences from the procedure reported by [1]. For a detailed description of the communication protocol between the participants and the main experimenter (GL), stimuli, and manipulation, please see the relevant sections in [1]. Preliminary results of the experiment were presented as a poster in the Conference on Human Factors in Computing Systems (CHI) 2022 [see [122]].

### Replication Framework

The aim of our replication study was to validate the findings of our previous work [1] by applying the same experimental setup to a different target population-caregivers. The original study focused on evaluating long-term self-disclosure for a general population, while the current study narrows the research question to examine how the behavioural paradigm introduced in [1] can be applied as an intervention supporting informal caregivers to cope with emotional distress over time. This approach allows us to test the robustness and generalisability of our original findings in a new context and with a dedicated population while assessing the paradigm as a potential intervention.

In replicating the experimental setup, we maintained methodological consistency to ensure the reliability and validity of our findings. This included the use of the same disclosure tasks, measurement tools, and analysis techniques to facilitate accurate comparisons between the two studies. However, several adjustments were made to tailor the study to the specific needs and characteristics of the intervention and target population (i.e., informal caregivers). These modifications included changes in the recruitment process to specifically target informal caregivers and the introduction of additional measurements focused on the emotional experiences of informal caregivers (e.g., caregiver burden [123]). Additionally, since we aimed to test if this behavioural paradigm could be applied as an intervention for a population in need, this study included a single condition, unlike the original study, which included two conditions [1]. Our previous findings indicated that manipulating the emotional frame of the questions or eliciting stress was unnecessary for this replication study. Finally, we included relevant measures to assess how this intervention could support emotion regulation among informal caregivers [124], focusing on their coping mechanisms and overall emotional well-being. Therefore, we could explore how self-disclosure towards robots helps informal caregivers change their perspective regarding their caregiving experience and cope better with the emotional distress that is associated with it.

Accordingly, this replication study’s results allow us to (a) further validate [1] previous results, (b) see how it can be applied as an intervention for a population in need, and (c) extend the original results with insights related to participants’ emotion regulation skills.

### Experimental Design

A one-way repeated measures experimental design with 10 repetitions (chat sessions across time) was conducted. Participants conversed with the robot Pepper (SoftBank Robotics) via Zoom video chats about general everyday topics (e.g., social relationships, work-life balance, health and well-being; see Sect. 3.5) for 10 sessions. Each interaction consisted of the robot asking the participant 3 questions (x3 repetitions). The topic of each interaction was assigned randomly before the experimental procedure started, as was the order of the questions. Participants were scheduled to interact with the robot twice a week during prearranged times for five weeks.

### Participants

#### Target Population

The target population for this study was exclusively adult (aged 18+) informal caregivers. These are adults from the general population aged 18 or over who are having extra responsibilities looking after a friend or a family member due to a long-term physical or mental ill-health or disability, or problem related to old age [24]. Moreover, participants reported having normal to corrected to normal vision, not suffering from hearing loss or difficulties, or physical handicap, are native English speakers, and currently reside in Great Britain. Due to the technical requirements of the mediated experimental design, the target population of this study consist of individuals with access to a personal computer with Zoom installed, a functioning web camera, a stable internet connection, a microphone, and speakers/headphones.

#### Recruitment

Participants were recruited via Prolific, restricted to those over 18, native English speakers, informal primary caregivers in the UK, with the necessary technical equipment. They committed to attending two sessions weekly for five weeks. Eligible Prolific users accessed the study page for detailed information and to complete the induction questionnaire on Qualtrics if interested. The induction introduced the study, tasks, and schedule, and required participants to confirm their commitment. Committed participants then selected their time slots and received a participant number. Participants were paid $\pounds$3 per 30-minute session and an additional $\pounds$20 upon completing all 10 sessions. Detailed recruitment procedures and specific Prolific filters are available on the study’s OSF page [see [121]].

#### Sample

A priori power calculations using G*power software [125, 126] suggest that for reasonable power (0.83) to detect small to medium effect sizes, a sample size of 22 participants would be required. Due to the relatively complex data collection procedure and the potential for a high dropout rate, we recruited 40 participants via the Prolific website. Two participants who were recruited for the study ended up not participating in any of the sessions. Additionally, throughout the study four more participants dropped out, mainly due to their caregiving responsibilities, resulting in a final sample size of 34 participants.

Participants were between the ages of 19 and 63 (*M* = 39.18, *SD* = 11.44), 67.6% identify as females, 29.4% identify as males, and 2.9% identify as non-binary/third gender. Half of the sample reports having a Bachelor’s degree as their highest level of education, and about a third (32.3%) reports lower educational qualifications. 44.1% are employed full-time, 11.8% employed part time, 23.5% are self-employed, and 20.6% are unemployed. Almost two-thirds (73.5%) of the sample are either married (44.1%) or in a relationship (29.4%). 50% of the sample have at least one child. Most of the participants (94.1%) did not live on their own during their participation in the study, with an average number of 3 individuals (*SD* = 1.47) in a household (including the participant). 41.2% of the sample consisted of participants who lived with only one additional person in their home other than themselves, and 47.1% of participants reported living with their care recipient. Almost all of the participants (85.3%) did not have previous experience with robots.

Most of the sample (91.2%) reported providing care to only one person, and more than half of the sample (58.8%) provide care to a partner or spouse. Almost 80% (79.4%) of the sample has been providing care for at least 3 years, with 29.4% of the sample reporting providing care for more than 5 years. The most common care recipients’ health condition reported is old age and related physical symptoms (e.g., mobility issues) and conditions (e.g., arthritis, osteoporosis) and related mental and cognitive symptoms (e.g., frailty, confusion and memory loss) and conditions (e.g., dementia). Other common conditions reported in the sample are mental health issues (including depression, anxiety, and complex post-traumatic stress disorder), autism, diabetes, heart failure, and cancer. Participants reported an average score of 9.94 (*SD* = 4.81) in terms of their care recipient functionality, with a minimum score of 1 and a maximum score of 22 (see Sect. 3.6.2). Carer participants reported a high subjective relationship perception score with their care recipient (*M* = 8, *SD* = 2.13), and an average of 5.52 (*SD* = 2.09) in terms of perceived care recipient responsiveness, with a minimum score of 1 and a maximum score of 9 (see Sect. 3.6.2).

#### Ethics and Communication

Study procedures were approved by the research ethics committee of the University of Glasgow (ethics approval number 300200132). All participants provided written informed consent before the study. Optional consent was sought for using their video and audio footage in research publications, conference presentations, and other multimedia outputs. Interested Prolific users were briefed about the study and its requirements but not about the functionalities of the robot Pepper to minimise any priming. In each session, including the induction session, participants were reintroduced to the study, reminded of their schedule, and informed about the study’s procedures. They were assured of the benefits and reminded that no risks were anticipated, and that they could withdraw at any time without penalty. Participants were informed about the use of their data, their right to withdraw it, and assured of privacy and anonymity. Contact information for the main researcher (GL) was provided for any follow-up questions. After the study, participants received a debriefing via Prolific, explaining the study and the use of the Wizard of Oz (WoZ) approach, and were given contact details for further questions or feedback. Further detailed information regarding the communication protocol between the participants and the main experimenter (GL) can be found in [1].

### Stimuli

**Fig. 1:**
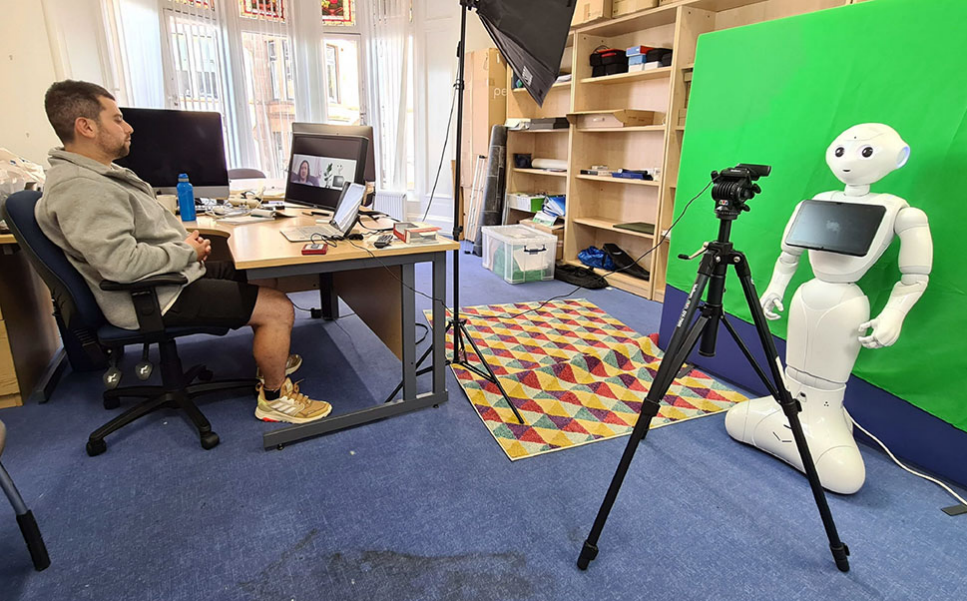
The lab settings, including the robot Pepper (SoftBank Robotics) in front of a web camera, while the experimenter in the back is controlling the robot using the Wizard of Oz technique

**Fig. 2:**
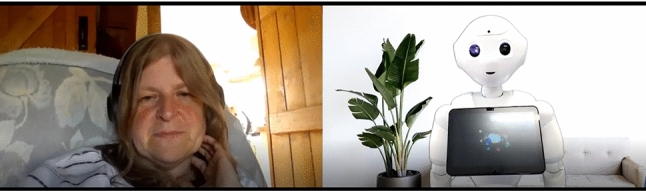
The interaction from the eyes of the participants and the experimenter. The participants were exposed only to the robot Pepper (SoftBank Robotics) via the zoom chats

Conversational interactions were guided by the humanoid robot Pepper (SoftBank Robotics), capable of communicating via speech and gestures. Following Leite’s (2013) guidelines for social robots in long-term interactions, Pepper was chosen for its suitable humanoid embodiment for the conversational task. Pepper, while human-like with a head, face, torso, arms, and hands, is not designed to resemble a real person. Instead, it communicates using human speech but lacks facial expressions due to its rigid face and head. Positioned in front of a Logitech 1080p web camera connected to the experimenter’s computer, Pepper had a green screen behaind it showcasing a white wall and a flowerpot (see Figs. 1 and 2). The semi-automatic WoZ technique controlled Pepper’s communication, with the experimenter using a PC laptop to trigger pre-scripted questions and speech items. Further detailed information regarding the stimuli and Pepper’s communication capabilities can be found in [1].

### Behavioural Paradigm

The paradigm is addressed in [1, 3.4 Behavioural Paradigm] and will only be briefly mentioned here. Each interaction was guided by Pepper (controlled by the experimenter in a WoZ set up) as a semi-structured interview discussing non-sensitive topics regarding general everyday experiences. Each interaction followed the same order, starting with greetings followed by 3 questions (x3 repetitions). The participants were instructed to have a short conversation with Pepper, following Pepper’s lead in the interaction and answering Pepper’s questions.The task followed the following structure and order:

- Short greetings/introduction (e.g., “Hi there, how are you doing?").
- One pre-defined general question about the participant’s day, week, or weekend, to build rapport (e.g., “how was your weekend? Did you do anything interesting?").
- An opening statement introducing the topic of the question (e.g., “I am about to ask you about your social life").
- Two pre-defined, non-sensitive questions that correspond to the topic that was randomly allocated to the interaction.

The questions and topics in the study were influenced by [16] and [127] as an elicitation technique aiming to capture participants’ subjective experiences regarding ten everyday topics [Work, Leisure and Passions, Finances, Relationships, Social Life, Mental Health, Physical Health, Personality, Goals and Ambitions, & Routine and Daily Activities; see [1]]. Here we used the same questions from the ’general everyday topics’ condition from [1]. Further detailed information regarding the manipulation and the task, including the task’s structure and content, can be found in [1].

### Measurements

To ensure that our models only include high-quality data, we included only cases that were captured and processed correctly.

#### Demographics

Participants were requested to complete a short questionnaire that gathered information on demographic parameters including age, biological sex, gender identification, level of education, nationality, job, previous experience with robots, and whether English is their native language.

#### Information Related to the Caregiving Situation

Questions concerning caregiving-related parameters included the length of time since they started providing care, how many people they provide care to, relationship to the care recipient, whether they live in the same house as their care recipient, their relationship quality with the care recipient, and the health condition of the care recipient. In addition, participants completed two additional scales:

*Perceived care recipient responsiveness*

This twelve item scale assesses how participants experienced and perceived their care recipient responsiveness using an adapted version of the perceived partner responsiveness scale by [128] to the caregiving situation. Each item concerning the informal caregiver perception of the care recipient is scored on a nine-point scale ranging from 1 (not at all true) to 9 (completely true). Accordingly, a mean scale was constructed ($M$ = 5.52, $SD$ = 2.09) which was found to be reliable (Cronbach’s $\alpha$ =.97).

*Care recipient functionality*

To assess the care recipient functionality and the intensity of care provided an adjusted version of Lawton’s Instrumental Activities of Daily Living [IADL; [129]] was employed. The scale includes 11 statements addressing different aspects of the care recipient’s daily functionality (e.g., taking a bath or a shower, preparing his/her own meals) that can be rated by the caregiver as 0 (without any help), 1 (with some help–person or device), or 2 (completely unable to perform the task independently). Accordingly, a sum scale was constructed ($M$ = 9.94, $SD$ = 4.81) which was found to be reliable (Cronbach’s $\alpha$ =.85).

#### Disclosure

*Subjective Self-Disclosure*

Participants were requested to report their level of subjective self-disclosure via an adaptation of the sub-scale of work and studies disclosure in Jourard’s Self-Disclosure Questionnaire [127]. This questionnaire was adapted to address disclosure in response to general life experiences, and to the context of the study (i.e., addressing specifically the participants’ disclosures to Pepper) [see [50]]. The measurement included ten self-reported items for which participants reported the extent to which they disclosed information to Pepper on a scale of one (not at all) to seven (to a great extent). Accordingly, a mean scale was constructed ($M$ = 3.42, $SD$ = 1.19) which was found to be reliable (Cronbach’s $\alpha$ =.84).

*Disclosure Duration*

Duration of speech in seconds from each recording was extracted and processed using Parselmouth [130], a Python library for Praat [131].

*Disclosure Length*

The volume of disclosure in terms of the number of words per disclosure. The recordings were automatically processed using the IBM Watson speech recognition engine, applying the British telephony model. To ensure capturing all utterances within each disclosure we amplified the audio files with 7 decibels and slowed the audio file’s pitch. The number of words per disclosure was extracted from the text using a simple length command in Python.

#### Perception

*Agency and Experience*

Research into mind perception has revealed that agency (the ability of an agent to plan and act) and experience (the ability of the agent to sense and feel) are two key dimensions when valuing an agent’s mind [132]. To determine whether any differences in mind perception emerged across the testing sessions, participants were requested to evaluate Pepper in terms of agency and experience, after being introduced to these terms [adapted from [132]]. Both concepts were evaluated by the participants using a 0 to 100 rating bar.

*Friendliness and Warmth*

The aim of this scale scale is to capture how participants perceived Pepper in terms of friendliness and warmth using one item from [133] and two items from [134], as suggested by [135]. These items were evaluated on a seven-point scale ranging from 1 (not at all) to 7 (extremely). Accordingly, a mean scale was constructed ($M$ = 5.92, $SD$ = 1.17) which was found to be reliable (Cronbach’s $\alpha$ =.95).

*Communication Competency*

This scale was aimed at capturing how participants experienced and evaluated Pepper’s communication competency using an adapted and adjusted version by [136] for a scale by [137]. The scale included three items that were evaluated on a seven-point scale ranging from 1 (not at all) to 7 (extremely). Accordingly, a mean scale was constructed ($M$ = 5.61, $SD$ = 1.19) which was found to be reliable (Cronbach’s $\alpha$ =.92).

*Interaction Quality*

This scale was aimed at capturing how participants perceived and evaluated the interaction with Pepper using an adapted and adjusted version by [136] for a scale by [138]. Each interaction included two random items out of seven, except for the mid-session (session 5) and the last session (session 10) which included all six items of the scale. These items were evaluated on a seven-point scale ranging from 1 (not at all) to 7 (extremely). Accordingly, a mean scale was constructed ($M$ = 5.11, $SD$ = 1.70) which was found to be reliable (Cronbach’s $\alpha$ =.96).

#### Well Being

*Mood*

To capture participants’ mood change from their interactions with Pepper, participants reported their mood before and after the interaction with Pepper using the Immediate Mood Scaler [IMS-12; see [139]]. IMS-12 includes 12 items of polarized moods, ranging from 1 (for negative moods) to 7 (for the corresponding positive moods). The scale is a novel validated tool based on the Positive and Negative Affect Schedule [PANAS; [140]], adapted and adjusted to capture current mood states in online and mobile experiments [139]. Mean reliable scales were constructed for participants’ mood before the interaction ($M$ = 4.90, $SD$ = 1.29, Cronbach’s $\alpha$ =.97) and after the interaction ($M$ = 5.24, $SD$ = 1.28, Cronbach’s $\alpha$ =.98).

*Comforting Responses*

To measure the extent to which participants perceived Pepper’s responses as comforting the comforting response scale was adapted from [141]. The scale includes 12 self-reported items rated on a seven-point scale, ranging from 1 (I strongly disagree) to 7 (I strongly agree). Accordingly, a mean scale was constructed ($M$ = 5.12, $SD$ =.82) which was found to be reliable (Cronbach’s $\alpha$ =.87).

*Loneliness*

In each session, participants were asked to report their feelings and thoughts of loneliness over the previous three days using the short-form UCLA loneliness scale [ULS-8; [142]]. The scale includes 8 items rated on a seven-point scale, ranging from 1 (not at all) to 7 (all the time). Accordingly, a mean scale was constructed ($M$ = 3.10, $SD$ = 1.68) which was found to be reliable (Cronbach’s $\alpha$ =.95).

*Stress*

Participants were requested to report their feelings and thoughts of periodic stress from the past month using the perceived stress scale [see [143]]. The scale includes 10 statement items rated on a seven-point scale, ranging from 1 (never) to five (very often). A mean scale was constructed ($M$ = 3.74, $SD$ = 1.46) which was found to be reliable (Cronbach’s $\alpha$ =.95).

*Caregiver Burden*

Participants were requested to evaluate statements addressing burdens associated with the caregiving experience using the short version of the Burden Scale for Family Caregivers [BSFC-s; [123]]. The scale includes 10 items for measuring subjective burden in informal caregivers. Each item is a statement that is rated on a 4-point scale with the values of (0) “strongly disagree”, (1) “disagree”, (2) “agree”, (3) and “strongly agree”. A high degree of agreement indicates a higher subjective burden for the caregiver. A sum scale was constructed ($M$ = 16.63, $SD$ = 6.69) which was found to be reliable (Cronbach’s $\alpha$ =.93).

#### Cognitive Emotion Regulation

In order to assess participants’ cognitive change and emotion regulation during the experiment and due to their interactions with Pepper, we used the short version of the Cognitive Emotion Regulation Questionnaire [CERQ-short; [124]] that is based on the original form of the Cognitive Emotion Regulation Questionnaire [CERQ; [144]]. The questionnaire includes 18 items addressing behaviours and thoughts that convey the practice of nine different strategies of coping and emotional regulation (2 items per technique) evaluated on a five-point scale, ranging from 1 (almost never) to five (almost always). The distinction between the nine strategies includes: Self-blame, Acceptance, Rumination, Positive refocusing, Refocus on planning, Positive reappraisal, Putting into perspective, Catastrophizing and Other-blame. A mean scale was constructed for each strategy, with a high score reflecting high use of the relevant behaviour or thought, with all of the conceptual scales showing good-high reliability except for ‘*Rumination*’ (moderate) and ‘*Refocus on planning*’ (low) (see Table 1).

**Table 1:** Mean, standard deviation and reliability scores of the cognitive emotion regulation sub-scales

**Strategy**	*M (SD)*	Cronbach’s $\alpha$
Self-Blame	1.87 (1.04)	0.83
Acceptance	4.20 (0.67)	0.76
Rumination	2.96 (0.91)	0.55
Positive refocusing	3.29 (0.95)	0.72
Refocus on planning	3.53 (0.73)	0.10
Positive reappraisal	3.55 (0.87)	0.71
Putting into perspective	3.53 (0.91)	0.70
Catastrophizing	2.40 (1.14)	0.88
Other-blame	2.01 (1.11)	0.89

### Materials

#### Zoom Video Chat

All interactions (video chats) were conducted with the software Zoom, using a university staff account (see Fig. 2). The interactions were recorded using the recording functionality on Zoom and edited to include only those portions of the recordings where participants and/or Pepper were speaking.

#### Qualtrics Questionnaires

All of the questionnaires were administered via the survey software Qualtrics, using a university staff account. In the online questionnaires, the functionality of recording participants’ IP addresses was disabled to comply with GDPR guidelines.

### Procedure

When recruited, participants completed an induction questionnaire (Session 0) about a week before beginning video chat interactions with Pepper (Sessions 1 to 10). They were instructed to have short conversations with Pepper on various everyday topics, with Pepper asking three questions per session, occurring twice a week for five weeks at prearranged times. Each interaction was to last 5-10 min, followed by 10-15 min for questionnaires. The induction questionnaire included instructions on video camera positioning and lighting, demographic questions, and several questionnaires. For the full list of questionnaires and their order in each session see the OSF repository [121]. Participants were redirected to Prolific after completing the induction questionnaire and were assigned a participant number for future sessions. The random allocation of topics and questions for each session was automated and recorded for each participant to help the experimenter control and follow the experimental design procedure for five weeks. See the randomization and allocation code, experimenter notebook with the allocated topics to sessions, and order of questions for each of the participants on the OSF repository [121].

At the start of each session (1 to 10), participants entered their Prolific ID and participant number, completed the Immediate Mood Scale [IMS-12; [139]], and received reminders and instructions for their interaction with Pepper. A Zoom link was provided, a frame with the zoom landing page, and the experimenter’s e-mail address and instructions on how to communicate with the experimenter in case there are any issues during the interaction. Participants interacted with Pepper via Zoom (see Sect. 3.5), seeing only Pepper in the chat (see Fig. 2). After the interaction, they returned to Qualtrics to complete the remaining questionnaires. The full list of questionnaires and their order in each session can be found on the study’s OSF page [see [121]]. Upon finishing, participants were thanked, reminded of their next session, given the experimenter’s contact details, and redirected to Prolific for a completion message. In the final session, participants were thanked for their participation, debriefed about the study, and provided with the experimenter’s contact details for any further questions.

## Results

### Disclosure

**Table 2:** Results of linear mixed effects analysis of session number effect on participants’ disclosure behaviour and perception outcomes

	**Subjective Disclosure**		**Duration**		**Length**	
**Fixed Effects**	*Estimates*	*95%CI*	*Estimates*	*95%CI*	*Estimates*	*95%CI*
Intercept	3.15$${^{***}}$$	2.78 - 3.52	16.95$${^{***}}$$	10.48 - 23.43	45.05$${^{***}}$$	27.49 - 62.61
Session Number	0.05$${^{***}}$$	0.03 - 0.08	2.78$${^{***}}$$	2.34 - 3.22	6.64$${^{***}}$$	5.45 - 7.83
**Random Effects**						
*SD*	0.99		17.47		47.40	
$\sigma ^{2}$	0.46		409.94		2985.92	
$\tau _{0 0}$	0.97		305.04		2247.18	
ICC	0.68		0.43		0.43	
N	34		34		34	
Observations	333		993		993	
Marginal $R^2$ / Conditional $R^2$	0.016 / 0.683		0.082 / 0.474		0.065 / 0.467	

*$$p<0.05$$ ** $$p<0.01$$ ***$$p<0.001$$

We used lme4 [145] for R to perform a linear mixed effects analysis of the effect of session number on participants’ disclosure to Pepper. We entered the session order as a fixed effect into the model. As a random effect, we had intercepts for subjects. Significance was calculated using the lmerTest package [146], which applies Satterthwaite’s method to estimate degrees of freedom and generate p-values for mixed models.

#### Subjective Self-Disclosure

**Fig. 3:**
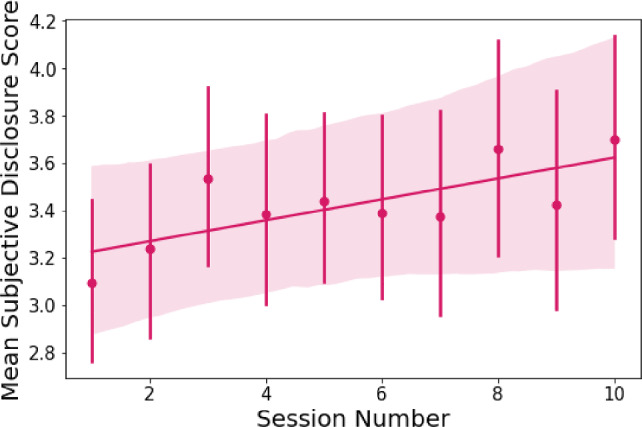
Mean subjective disclosure scores [adapted from [127], following [50]] by session number. The session number has a significant positive fixed effect on participants’ subjective perceptions of their self-disclosures. Therefore, participants perceived to be self-disclosing more to the social robot Pepper over time

The model explains 68.3% of the variance in participants’ subjective self-disclosure, whereas the fixed effect in the model explains 1.6% of the variance. The results stress that despite the variance between the participants ($SD$ =.99), the session number has a significant positive fixed effect on participants’ subjective perceptions of their self-disclosures ($\beta$ =.05, *SE* =.01, $p <.001$, see Table 2). Therefore, participants perceived to be self-disclosing more to the social robot over time (see Fig. 3).

#### Disclosure Duration

**Fig. 4:**
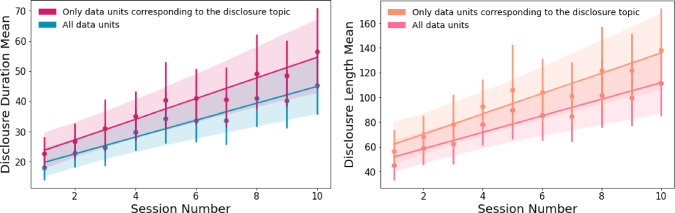
From left to right: **(1)** Mean disclosure duration (in seconds) by session number. In navy blue, all data units, in purple, only data units corresponding to the disclosure topic. Both lines indicate that the session number has a significant positive fixed effect on participants’ disclosure duration. Hence, participants self-disclosed increasingly more (in terms of duration in seconds) to the social robot Pepper over time. **(2)** Mean disclosure length (in number of words) by session number. In pink, all data units, in orange, only data units corresponding to the disclosure topic. Both lines indicate that the session number has a significant positive fixed effect on participants’ disclosure length. Hence, participants self-disclosed increasingly more (in terms of number of words) to the social robot Pepper over time

The model explains 47.5% of the variance in participants’ disclosure duration (in seconds) to Pepper, whereas the fixed effect in the model explains 8.1% of the variance in participants’ disclosure duration. The results stress that despite the variance between the participants ($SD$ = 17.63), the session number has a significant positive fixed effect on participants’ disclosures duration ($\beta$ = 2.78, *SE* =.23, $p <.001$, see Table 2). Hence, participants self-disclosed increasingly more (in terms of duration in seconds) to Pepper over time (see Fig. 4).

Another linear mixed effects model was used to test if the session number significantly predicted the disclosure duration when interacting with the social robot Pepper, including only the items corresponding to the disclosure topic. The model explains 60.3% of the variance in participants’ disclosures duration (in seconds) to Pepper, whereas the fixed effect in the model explains 10.2% of the variance. The results stress that despite the variance between the participants ($SD$ = 21.77), the session number has a significant positive fixed effect on participants’ disclosures duration ($\beta$ = 3.41, *SE* =.26, $p <.001$, see Table 3). Therefore, participants self-disclosed increasingly more (in terms of duration in seconds) to Pepper over time (see Fig. 4).

**Table 3:** Results of linear mixed effects analysis of session number effect on participants’ disclosure behaviour outcomes with only data units corresponding to the disclosure topic

	**Duration**		**Length**	
**Fixed Effects**	*Estimates*	*95%CI*	*Estimates*	*95%CI*
Intercept	20.30$${^{***}}$$	12.38 - 28.22	53.75$${^{***}}$$	32.20 - 75.29
Session Number	3.42$${^{***}}$$	2.90 - 3.93	8.18$${^{***}}$$	6.77 - 9.60
**Random Effects**				
*SD*	21.55		58.54	
$\sigma ^{2}$	371.54		2802.87	
$\tau _{0 0}$	464.27		3426.64	
ICC	0.56		0.55	
N	34		34	
Observations	662		662	
Marginal $R^2$ / Conditional $R^2$	0.104 / 0.602		0.082 / 0.587	

*$$p<0.05$$ **$$p<0.01$$ ***$$p<0.001$$

#### Disclosure Length

The model explains 46.9% of the variance in participants’ disclosures length (in number of words) to Pepper, whereas the fixed effect in the model explains 6.4% of the variance in participants’ disclosures length. The results stress that despite the variance between the participants ($SD$ = 47.92), the session number has a significant positive fixed effect on participants’ disclosures length ($\beta$ = 6.63, *SE* =.61, $p <.001$, see Table 2). Hence, participants self-disclosed increasingly more (in terms of number of words) to Pepper over time (see Fig. 4).

Another linear mixed effects model was used to test if the session number significantly predicted the disclosure length when interacting with the social robot Pepper, including only the items corresponding to the disclosure topic. The model explains 58.9% of the variance in participants’ disclosures length (in number of words) to Pepper, whereas the fixed effects in the model explain 8% of the variance. The results stress that despite the variance between participants ($SD$ = 59.21), session number has a significant positive fixed effect on participants’ disclosures length ($\beta$ = 8.17, *SE* =.73, $p <.001$, see Table 3). Hence, participants self-disclosed increasingly more (in terms of number of words) to Pepper over time (see Fig. 4).

### Perception

We used lme4 [145] for R to perform linear mixed effects analysis of the effect of session number on participants’ perceptions of Pepper, including perceptions of agency and experience [see [132]], friendliness and warmth, communication competency and interaction quality. We entered the session order as a fixed effect. As a random effect, we had intercepts for subjects. Significance was calculated using the lmerTest package [146], which applies Satterthwaite’s method to estimate degrees of freedom and generate p-values for mixed models.

#### Agency

**Fig. 5:**
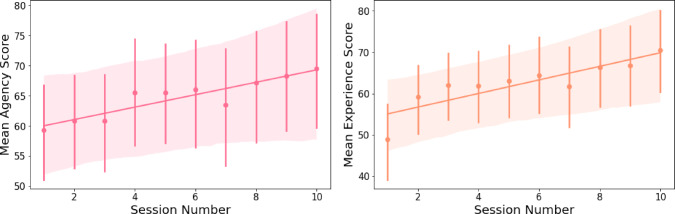
From left to right: **(1)** Mean scores of agency [i.e., the ability of the agent to plan and act; see [132]] by session number. The session number has a significant positive fixed effect on participants’ perceptions of the social robot Pepper’s degree of agency. Therefore, participants perceived the social robot Pepper to demonstrate higher degrees of agency over time. **(2)** Mean scores of experience [i.e., the ability of the agent to sense and feel; see [132]] by session number. The session number has a significant positive fixed effect on participants’ perceptions of the social robot Pepper’s degree of experience. Therefore, participants perceived the social robot Pepper to demonstrate higher degrees of experience over time

The model explains 82.4% of the variance in participants’ perceptions of Pepper’s degree of agency, whereas the fixed effect in the model explains 1.3% of the variance. The results stress that despite the variance between the participants ($SD$ = 23.52), the session number has a significant positive fixed effect on participants’ perceptions of Pepper’s degree of agency ($\beta$ = 1.03, *SE* =.21 $p <.001$, see Table 4). Therefore, participants perceived Pepper to demonstrate higher degrees of agency over time (see Fig. 5).

**Table 4:** Results of linear mixed effects analysis of session number effect on participants’ social perceptions of Pepper

	**Agency**		**Experience**		**Friendliness and Warmth**	
**Fixed Effects**	*Estimates*	*95%CI*	*Estimates*	*95%CI*	*Estimates*	*95%CI*
Intercept	59.06$${^{***}}$$	50.73 - 67.40	53.42$${^{***}}$$	44.73 - 62.10	5.56$${^{***}}$$	5.18 - 5.93
Session Number	1.03$${^{***}}$$	0.62 - 1.45	1.67$${^{***}}$$	1.20 - 2.13	0.07$${^{***}}$$	0.05 - 0.08
**Random Effects**						
*SD*	23.52		24.29		1.07	
$\sigma ^{2}$	120.24		152.73		0.20	
$\tau _{0 0}$	553.28		590.05		1.14	
ICC	0.82		0.79		0.85	
N	34		34		34	
Observations	333		333		333	
Marginal $R^2$ / Conditional $R^2$	0.013 / 0.824		0.030 / 0.801		0.026 / 0.852	

*$$p < 0.05$$ **$$p< 0.01$$ ***$$p < 0.001$$

#### Experience

The model explains 80.1% of the variance in participants’ perceptions of Pepper’s degree of experience, whereas the fixed effect in the model explains 3% of the variance. The results stress that despite the variance between the participants ($SD$ = 24.29), the session number has a significant positive fixed effect on participants’ perceptions of Pepper’s degree of experience ($\beta$ = 1.67, *SE* =.24, $p <.001$, see Table 4). Therefore, participants perceived Pepper to demonstrate higher degrees of experience over time (see Fig. 5).

#### Friendliness and Warmth

The model explains 85.2% of the variance in participants’ perceptions of Pepper’s degree of friendliness and warmth, whereas the fixed effect in the model explains 2.6% of the variance. The results stress that despite the variance between the participants ($SD$ = 1.07), the session number has a significant positive fixed effect on participants’ perceptions of Pepper’s degree of friendliness and warmth ($\beta$ =.07, *SE* =.01, $p <.001$, see Table 4). Therefore, participants perceived Pepper as friendlier and warmer over time (see Fig. 5).

#### Communication Competence

The model explains 72% of the variance in participants’ perceptions of Pepper’s communication competence, whereas the fixed effect in the model explains 3.5% of the variance. The results stress that despite the variance between the participants ($SD$ = 1), the session number has a significant positive fixed effect on participants’ perceptions of Pepper’s communication competence ($\beta$ =.08, *SE* =.01, $p <.001$, see Table 5). Therefore, participants perceived Pepper’s to be more competent over time.

**Table 5:** Results of linear mixed effects analysis of session number effect on participants’ usability-related perceptions of Pepper

	**Communication Competency**		**Interaction Quality**	
**Fixed Effects**	*Estimates*	*95%CI*	*Estimates*	*95%CI*
Intercept	5.19$${^{***}}$$	4.83 - 5.55	4.56$${^{***}}$$	4.05 - 5.07
Session Number	0.08$${^{***}}$$	0.05 - 0.10	0.10$${^{***}}$$	0.06 - 0.14
**Random Effects**				
*SD*	0.99		1.36	
$\sigma ^{2}$	0.40		0.96	
$\tau _{0 0}$	0.97		1.85	
ICC	0.71		0.66	
N	34		34	
Observations	333		333	
Marginal $R^2$ / Conditional $R^2$	0.035 / 0.720		0.030 / 0.669	

*$$p< 0.05$$ **$$p < 0.01$$ ***$$p< 0.001$$

**Table 6:** Results of linear mixed effects analysis of session number and mood change on participants’ well being

	**Mood**		**Comforting Responses**		**Loneliness**	
**Fixed Effects**	*Estimates*	95%CI	*Estimates*	95%CI	*Estimates*	95%CI
Intercept	4.94$${^{***}}$$	4.54 - 5.35	4.78$${^{***}}$$	4.53 - 5.03	3.31$${^{***}}$$	2.77 - 3.85
Session Number	$$-$$0.01	$$-$$0.03 - 0.01	0.06$${^{***}}$$	0.05 - 0.08	$$-$$0.05$${^{***}}$$	$$-$$0.06 - $$-$$0.02
Mood change	0.24${^{*}}$	0.05 - 0.43				
Session number * Mood change	0.02	$-$0.01 - 0.05				
**Random Effects**						
*SD*	1.15		0.67		1.57	
$\sigma ^{2}$	0.34		0.22		0.37	
$\tau _{0 0}$	1.32		0.43		2.46	
ICC	0.79		0.66		0.87	
N	34		34		34	
Observations	666		333		367	
Marginal $R^2$ / Conditional $R^2$	0.018 / 0.796		0.049 / 0.681		0.007 / 0.871	

*$$p< 0.05$$ **$$p< 0.01$$ ***$$p<0.001$$

#### Interaction Quality

The model explains 66.9% of the variance in participants’ perceptions of the interaction quality, whereas the fixed effect in the model explains 3% of the variance. The results stress that despite the variance between the participants ($SD$ = 1.36), the session number has a significant positive fixed effect on participants’ perceptions of the interaction quality ($\beta$ =.10, *SE* =.02, $p <.001$, see Table 5). Hence, participants perceived the interections with Pepper to be of higher quality over time.

### Well-being

We used lme4 [145] for R to perform a linear mixed effects analysis of the effect of session number on participants’ perceptions of Pepper’s comforting responses, mood change, feelings of loneliness and stress, and burdens from the caregiving experience. We used session order as a fixed effect in the model. As a random effect, we had intercepts for subjects. Significance was calculated using the lmerTest package [146], which applies Satterthwaite’s method to estimate degrees of freedom and generate p-values for mixed models.

#### Mood

**Fig. 6:**
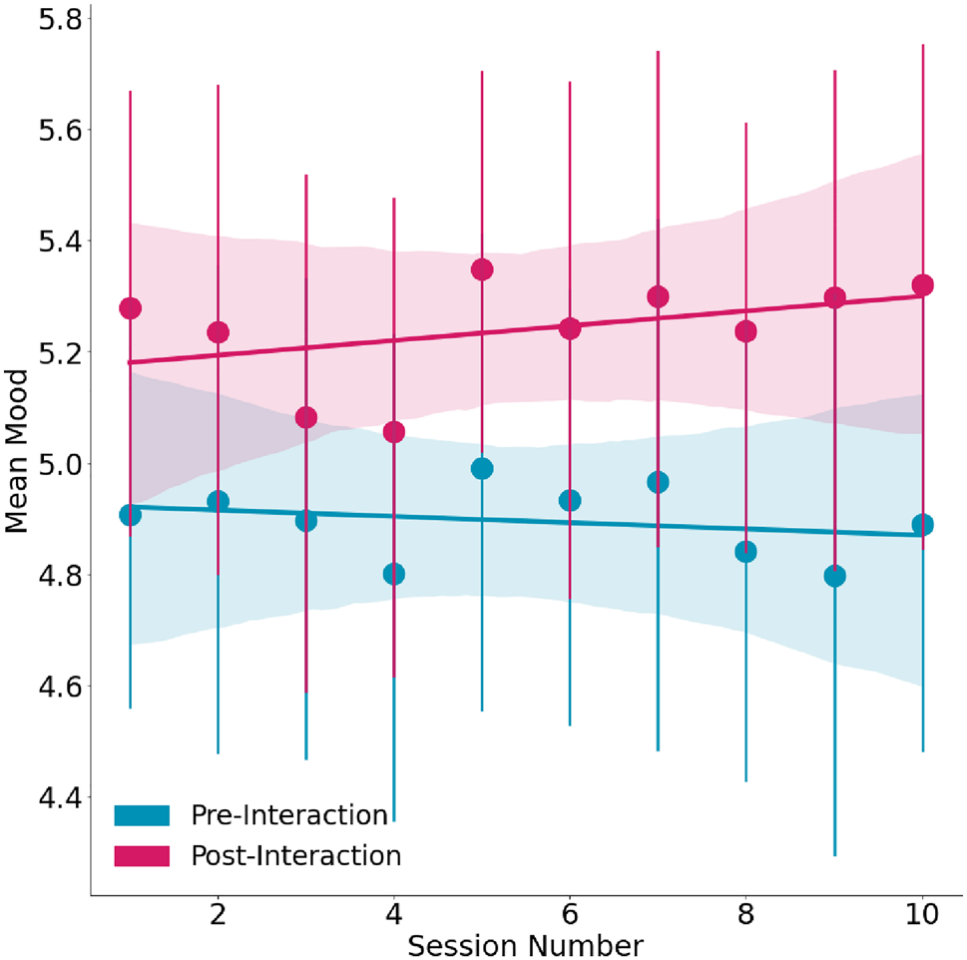
Mean mood scores [via IMS-12; see [139]] of participants before (in navy blue) and after (in purple) the interaction with the social robot Pepper by session number. The results indicate a positive significant fixed effect on mood change, as participants reported a positive mood change after interacting with Pepper. Therefore, participants’ mood improved after interacting with Pepper

The model explains 79.6% of the variance in participants’ mood, whereas the fixed effect in the model explains 1.8% of the variance. The results stress that despite the variance between the participants ($SD$ = 1.15), we observed a positive significant fixed effect on mood change, as participants reported a positive mood change after interacting with Pepper ($\beta$ =.24, *SE* =.01, $p =.014$, see Table 6). Therefore, participants’ mood improved after interacting with Pepper (see Fig. 6). Nevertheless, there were no significant fixed effects in terms of the session number ($\beta$ = -.01, *SE* =.01, *p* =.479), and the interaction term of mood change and the session number ($\beta$ =.02, *SE* =.02, *p* =.233, see Table 6).

**Fig. 7:**
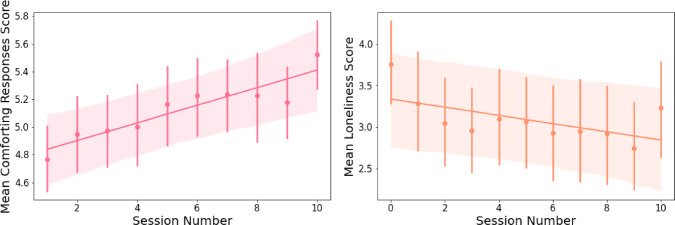
From left to right: **(1)** Mean comforting responses scores [adapted from [141]] by session number. The session number has a significant positive fixed effect on participants’ perceptions of Pepper’s comforting responses. Therefore, participants perceived Pepper’s responses to be more comforting over time. **(2)** Mean scores of loneliness [via ULS-8; see [142]] by session number. The session number has a significant negative fixed effect on participants’ feelings of loneliness. Hence, participants reported feeling less lonely over time

#### Comforting Responses

The model explains 68.1% of the variance in participants’ perceptions of Pepper’s comforting responses, whereas the fixed effect in the model explains 4.9% of the variance. The results stress that despite the variance between the participants ($SD$ =.66), the session number has a significant positive fixed effect on participants’ perceptions of Pepper’s comforting responses ($\beta$ =.06, *SE* =.01, $p <.001$, see Table 6). Therefore, participants perceived Pepper’s responses to be more comforting over time (see Fig. 7).

#### Loneliness

The model explains 87.1% of the variance in participants’ feelings of loneliness, whereas the fixed effect in the model explains 0.7% of the variance. The results stress that despite the variance between the participants ($SD$ = 1.57), the session number has a significant negative fixed effect on participants’ feelings of loneliness ($\beta$ = -.05, *SE* =.01, $p <.001$, see Table 6). Hence, participants reported feeling less lonely over time (see Fig. 7).

#### Stress

**Fig. 8:**
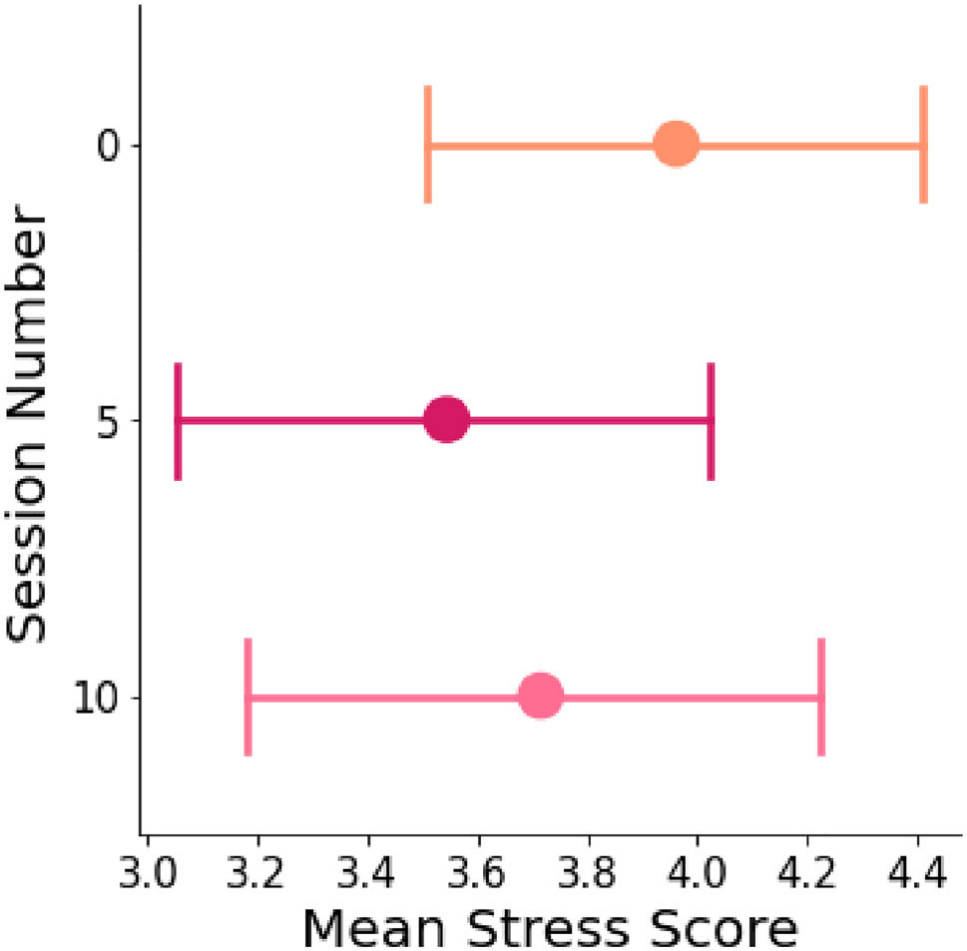
Mean stress scores [adapted from [143]] in sessions 0, 5, and 10. The session number has significant negative fixed effects on participants’ feelings of stress in the fifth session compared to the induction session and the last session, and in the last session compared to the induction session and the fifth session. Hence, participants reported feeling less stressed in the fifth session and the tenth session compared to the induction session, which was before engaging in the intervention. As such, the results reflect that participants experienced decreasing feelings of stress over time

The model explains 90.9% of the variance in participants’ feelings of stress, whereas the fixed effects in the model explain 1.4% of the variance. The results stress that despite the variance between the participants ($SD$ = 1.39), the session number has significant negative fixed effects on participants’ feelings of stress in the fifth session compared to the induction session and the last session ($\beta$ = −.42, *SE* =.11, $p <.001$), and in the last session compared to the induction session and the fifth session ($\beta$ = -.24, *SE* =.11, $p =.033$, see Table 7). Hence, participants reported feeling less stressed in the fifth session and the tenth session compared to the induction session, which was before engaging in the intervention. As such, the results reflect that participants experienced decreasing feelings of stress over time (see Fig. 8). However, it is important to highlight that while stress seems to decrease over time, the lowest reported stress was mid-study, with no significant differences between perceived stress measured on session 5 and session 10. With only three data points of perceived stress throughout the study (in the induction session, session 5, and the last session), it would be valuable for longer-term interventions to examine the relationship between prolonged interactions with social robots and stress perceptions in more detail, and potentially using objective physiological measures [see [147]].

**Table 7:** Results of linear mixed effects analysis of session number effect on participants’ perceived stress

	**Stress**	
**Fixed Effects**	*Estimates*	*95%CI*
Intercept	3.96$${^{***}}$$	3.47 - 4.46
Session Number Five	$$-$$0.42$${^{***}}$$	$$-$$0.63 $$-$$0.21
Session Number Ten	$-$0.24${^{*}}$	$-$0.45 $-$0.02
**Random Effects**		
*SD*	1.39	
$\sigma ^{2}$	0.19	
$\tau _{0 0}$	1.92	
ICC	0.91	
N	34	
Observations	101	
Marginal $R^2$ / Conditional $R^2$	0.014 / 0.909	

*$$p< 0.05$$ **$$p<0.01$$ ***$$p< 0.001$$

#### Caregiver Burden

The model explains 84.5% of the variance in participants’ subjective perceptions of burden from their caregiving experience, whereas the fixed effect in the model does not explain any of the variance. The results stress that while considering for the variance between the participants ($SD$ = 6.18), the session number was not found to be a significant predictor for change in participants’ perceptions of burden from caregiving. We found no significant difference between participants’ burden scores in the induction and the last sessions ($\beta$ =.23, *SE* =.65, $p =.727$). Hence, there is no effect of repeated interactions self disclosing to the social robot Pepper on informal caregiver burden.

### Cognitive Emotion Regulation

**Table 8:** Results of linear mixed effects analysis of session number effect on participants’ adaptation of cognitive emotion regulation strategies

**Fixed Effects of Session Number on:**	*Estimates (SE)*	*p*
Self-Blame	$-$0.29 (0.17)	0.105
Acceptance	**0.30 (0.13)**	**0**.**024**
Rumination	$-$0.22 (0.16)	0.168
Positive refocusing	0.20 (0.15)	0.174
Refocus on planning	$-$0.10 (0.16)	0.553
Positive reappraisal	**0.30 (0.15)**	**0**.**045**
Putting into perspective	0.60 (0.15)	0.699
Catastrophizing	$-$0.19 (0.16)	0.249
Other-blame	$-$ **0.28 (0.14)**	**0**.**047**

’Session number’ is a dummy variable of sessions 0 and 10 (10 = 1)

We used lme4 [145] for R to perform a linear mixed effects analysis of the effect of session number on participants’ cognitive emotion regulation strategies, including self-blame, acceptance, putting into perspective, rumination, positive refocusing, positive reappraisal, catastrophizing, blaming others, and refocusing on planning. We used session order as a fixed effect in the model, used as a dummy variable of sessions 0 and 10 whereas 0 is the reference group. As a random effect, we had intercepts for subjects. Significance was calculated using the lmerTest package [146], which applies Satterthwaite’s method to estimate degrees of freedom and generate p-values for mixed models. We performed 9 models for the 9 strategies. Six of the models do not explain any of the variance in participants’ cognitive emotion regulation strategies adaption from session 0 to session 10, including self-blame, rumination, positive refocusing, refocus on planning, putting into perspective, and catastrophizing (see Table 8). Three of the models provide support to positive adaptation of three of the strategies, including acceptance, positive reappraisal, and other-blame (see Tables 8 and 9).

**Table 9:** Results of linear mixed effects analysis of session number effect on participants’ acceptance, positive reappraisal, and other blame

	**Acceptance**		**Positive Reappraisal**		**Other-Blame**	
**Fixed Effects**	*Estimates*	95%CI	*Estimates*	95%CI	*Estimates*	*95%CI*
Intercept	4.12$${^{***}}$$	3.90 - 4.34	3.43$${^{***}}$$	3.13 - 3.72	2.10$${^{***}}$$	1.73 - 2.48
Session Number	0.30${^{*}}$	0.05 - 0.55	0.30${^{*}}$	0.01 - 0.59	$-$0.28${^{*}}$	$-$0.55 - $-$0.01
**Random Effects**						
*SD*	0.38		0.63		0.94	
$\sigma ^{2}$	0.27		0.35		0.31	
$\tau _{0 0}$	0.14		0.40		0.88	
ICC	0.35		0.53		0.74	
N	34		34		34	
Observations	67		67		67	
Marginal $R^2$ / Conditional $R^2$	0.053 / 0.385		0.030 / 0.546		0.016 / 0.747	

*p<0.05 ** p<0.01 *** p<*0.001*

*’Session number’ is a dummy variable of sessions 0 and 10 (10 = 1).*

**Fig. 9:**
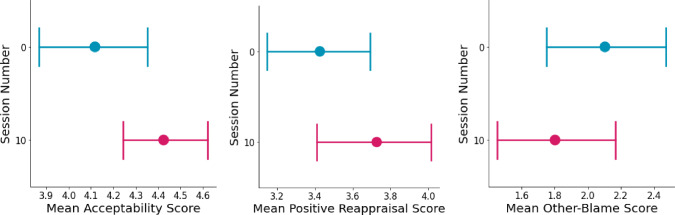
From left to right: **(1)** Mean score of acceptance [see [124]] in session 0 and session 10. The session number has a significant positive fixed effect on participants’ acceptance of the caregiving situation. In other words, participants were more accepting of their caregiving situation after participating in the intervention. **(2)** Mean score of positive reappraisal [see [124]] in session 0 and session 10. The session number has a significant positive fixed effect on participants’ adaption of positive reappraisal of the caregiving situation. In other words, participants reappraised their caregiving experience more positively after participating in the intervention. **(3)** Mean score of other-blame [see [124]] in sessions 0 and 10. The session number has a significant negative fixed effect on participants’ tendency to blame others for their caregiving situation. In other words, participants reported experiencing fewer feelings of blame towards others

#### Acceptance

The model explains 38.5% of the variance in participants’ change in acceptance of the caregiving situation from the induction session (session 0) to the last session (session 10), whereas the fixed effect in the model explains 5.3% of the variance. The results stress that despite the variance between the participants ($SD$ =.38), the session number has a significant positive fixed effect on participants’ acceptance of the caregiving situation ($\beta$ =.30, *SE* =.13, $p =.024$, see Table 9). In other words, participants were more accepting of their caregiving situation after participating in the intervention (see Fig. 9).

#### Positive Reappraisal

The model explains 54.6% of the variance in participants’ adaption of positive reappraisal of the caregiving situation from the induction session (session 0) to the last session (session 10), whereas the fixed effect in the model explains 3% of the variance. The results stress that despite the variance between the participants ($SD$ =.63), the session number has a significant positive fixed effect on participants’ adaption of positive reappraisal of the caregiving situation ($\beta$ =.30, *SE* =.15, $p =.045$, see Table 9). In other words, participants reappraised their caregiving experience more positively after participating in the intervention (see Fig. 9).

#### Other-Blame

The model explains 74.7% of the variance in participants’ tendency to blame others for their caregiving situation from the induction session (session 0) to the last session (session 10), whereas the fixed effect in the model explains 1.6% of the variance. The results stress that despite the variance between the participants ($SD$ =.94), the session number has a significant negative fixed effect on participants’ tendency to blame others for their caregiving situation ($\beta$ = -.28, *SE* =.14, $p =.047$, see Table 9). In other words, participants reported experiencing fewer feelings of blame towards others (see Fig. 9).

## Discussion

We have introduced a novel long-term mediated intervention aimed at supporting informal caregivers to cope with emotional distress via self-disclosing their emotions and needs to a social robot. This study was a replication of [1] with informal caregivers, a population that is normally coping with emotional distress [20, 28]. We were specifically interested in the extent of informal caregivers’ self-disclosure behaviour towards the robot over time and how these people’s perceptions of the robot developed over time. Moreover, we were interested in how this intervention impacted informal carers’ mood, feelings of loneliness, and stress, as well as how it affected their cognitive emotion regulation strategies and thoughts. Participants conversed with the social robot Pepper 10 times over 5 weeks about general everyday topics. Our results replicated [1] showing that informal caregivers self-disclose increasingly more to a social robot over time (in terms of disclosure duration in seconds, and disclosure length in number of words, and also in terms of informal caregivers’ subjective perceptions of their own disclosures to Pepper), and perceive the robot as more social and competent over time. Furthermore, we found that informal caregivers’ moods positively change after interacting with the robot, they also perceive the robot to be more comforting over time, and they feel less lonely and stressed overtime during their participation in the intervention. Finally, our results revealed that after participating in the intervention and self-disclosing to Pepper for 5 weeks, informal caregivers reported being more accepting of their caregiving situation, reappraising it more positively, and experiencing lower feelings of blame towards others.

The present findings replicate our previous result [1], providing compelling evidence for social robots’ potential to establish meaningful relationships with human users. While consistent with previous suggestions on the matter [see [148, 149]], the present study, together with [1], provides initial support for long-term relationships between human users and social robots, supported by multidimensional data. These findings support the establishment of important foundations for future HRI studies examining how human-robot relationships develop over time, as well as crucial theory for roboticists aiming to develop meaningful relationships between their robots and users. Taken together, these findings could support the introduction of social robots in well-being settings and as interventions supporting individuals in need.

**Table 10:** Summary of key research themes and objectives, including measurements collected and key results

**Key Themes & Objectives**	**Measurements Collected**	**Evidence from Results**
Self-disclosure behaviour (O1)	$\bullet$ Duration (in seconds)	Participants gradually disclosed more information to Pepper over time (see Sects. 4.1.2 and 4.1.3) and also reported perceiving themselves as sharing more with Pepper as time went on (see Sect. 4.1.1)
	$\bullet$ Length (number of words)	
	$\bullet$ Subjective self-disclosure [127]	
Social perception and usability (O2)	$\bullet$ Agency [the robot ability to plan and act; [132]]	Participants perceived Pepper as increasingly social (in terms of agency, and friendliness and warmth) and competent (in terms of communication competency and interaction quality) over time (see Sect. 4.2)
	$\bullet$ Experience [the robot ability to sense and feel; [132]]	
	$\bullet$ Friendliness and warmth [133, 134]	
	$\bullet$ Communication competency [136, 137]	
	$\bullet$ Interaction quality [136, 138]	
Emotional well-being (O3)	$\bullet$ Mood [IMS-12; [139]]	Participants’ moods positively changed after interacting with Pepper (see Sect. 4.3.1), which they perceived more comforting over time (see Sect. 4.3.2). Participants also reported feeling increasingly less lonely and stressed (see Sects. 4.3.3 and 4.3.4). There is no evidence that the intervention affected participants’ perception of the burden related to their caregiving experience (see Sect. 4.3.5)
	$\bullet$ Comforting responses [141]	
	$\bullet$ Loneliness [142]	
	$\bullet$ Stress [143]	
	$\bullet$ Caregiver burden [123]	
Cognitive changes in emotion regulation (O4)	$\bullet$ Cognitive Emotion Regulation Questionnaire [CERQ; [124]]	Participants reported being more accepting of their caregiving situation, reappraising it more positively, and experiencing fewer feelings of blame towards others (see Sect. 4.4)

### Robot-led Interactions for Emotionally Distressed Individuals

Self-disclosure is a dynamic and socially complex human behaviour [16, 17], and accordingly, the present findings contribute to the understanding of humans’ social behaviour and communication with robots. The results of this study complement previous studies addressing humans’ social behaviour towards robots in prolonged interactions [e.g., [1, 107]], and emphasise how people people open up and socially share with robots. This finding is particularly relevant considering that participants’ behaviours in the present study corresponded to their subjective social perceptions of the robot. These findings thus contribute to HRI research and theory by showing users’ self-disclosure behavioural changes toward social robots during prolonged and intensive interactions from objective behavioural evidence, as well as from users’ self-reported subjective perceptions.

Despite Pepper’s limited responses, over time participants attributed more social qualities to this particular robot, thus providing evidence for the influence of social engagement with a robot on its social perception over time. Furthermore, beyond finding Pepper to be more social, participants also attributed higher degrees of competency to Pepper over time. Due to the context of the study and the nature of the task, this provides the research community with substantial evidence for the feasibility and adaptation of social robots as interventions. The current state of technology is still far from enabling an open dialogue between humans and robots for establishing relationships, at least until Large Language Models facilitate social interactions in embodied agents [150]. However, our results demonstrate how adaptive human users’ perceptions and behaviours can be during relationship formation efforts with a social robot. In other words, while robots are not yet adaptive towards humans, our findings, along with those in [1, 114], indicate that human psychology can overcome this limitation. Over time, humans attribute meaning to interactions with robots, facilitating richer and more meaningful engagements. It is important to consider that the interactions in this study were short and restricted to specific limited domains. Accordingly, we can assume that users’ expectations of robots are fulfilled when HRIs are aimed at answering specific needs in a specific setting, and therefore, they find the robot to be social and competent over time despite the robot’s limitations and limited set of responses.

Finally, considering the positive outcomes on participants well-being due to their disclosures to the robot, these results highlight how human–robot relationships could act as ideal settings for robotic interventions for well-being. These results are particularly interesting due to the unique life situation of this study’s target population, informal caregivers [see [24]]. These individuals are under significant emotional distress and deal with many complex burdens [20, 27, 28]. Accordingly, from the results of this study, we can learn about the value of social robot-led interactions with emotionally distressed individuals who might not be suffering from a diagnosed mental condition or illness themselves, but who are living with considerably difficult life situations. Social robots could therefore be used as a tool to facilitate social interactions with emotionally distressed individuals over time, acquire relevant information from their disclosures, and potentially relieve their stress and burden via engaging them in ongoing discussions that elicit rich self-disclosures. We aspire to see more research and development in HRI and social robotics invested into supporting informal caregivers and not only care recipients.

We suspect that while interventions aimed at providing practical assistance (e.g., provision of information about health conditions, care planning, advice about patient management, skills training to aid patient management, stress management training, and problem-solving and decision-making guidance) to informal caregivers to reduce burden [see [151]] would have a direct effect on informal caregivers’ perceptions of caregiving burden, interventions that are oriented towards providing emotional support to informal caregivers (like the one reported in this paper) would have *indirect* (i.e., *mediating*) effect on caregiving burden via caregivers’ mood, stress, and other factors related to their emotional well-being. Therefore, despite that our results did not find support for the intervention affecting perceptions of caregiving burden, we would like to encourage further investigation of how interpersonal regulation interventions with social robots and other emerging technologies can support the feelings of burden among caregivers due to the tremendous effect it has on informal caregivers’ life and well-being [see [29, 30]].

### Communicating with Robots to Avoid Suppression of Self-Disclosure

The findings of the study highlight the capacity of social robots to elicit meaningful and rich self-disclosure from people, including informal carers (the participant population for the current study), who are people under a considerable amount of emotional distress. Suppressive behaviour of self-disclosure is maladaptive [9], associated with symptoms of depression [152], and might impact informal caregivers’ emotional well-being in a variety of ways [75]. This behaviour is common among informal caregivers [see [68, 71–73]] and can drastically impact an informal caregiver’s symptoms of depression and anxiety [76]. Our results provide objective behavioural evidence for a positive behaviour change in terms of self-disclosure. Throughout the study period, Pepper elicited richer disclosures from the informal caregivers and reduced the potential levels of suppression accordingly.

More explicitly, our results here show the potential of these interactions to reduce suppression via the elicitation of higher rates of self disclosure over time (in terms of quantities, and with the additional support of participants’ perceptions of their own disclosures), as higher quantities of self-disclosure is positively associated with more intimate and personal self-disclosure [153, 154]. Nonetheless, our data is currently limited from qualitatively describing the relevancy of participants’ disclosures to the caregiving experience and related stressors. The study results are limited from showing that the self-disclosure behaviour practised in this study objectively reduced suppressive behaviour that is related to the emotional distress experienced by informal caregivers. In other words, we do not know if the informal caregivers that participated in this study avoided self disclosing matters that are related to their caregiving experience, and shared with Pepper information that is corresponding to their caregiving experience. However, while we might expect informal caregivers to mention the caregiving experience (or care-related themes in general) when avoiding suppression [c.f., [69, 70]], many informal caregivers (or, people who are emotionally distressed in general) would like to be disclosing (and conversing, in general) about variety of matters that are more directly related to their emotions and feelings (that might not be related to the caregivng experience). This is particularly important in the context of informal caregivers, considering that the caregiving experience takes such a big part of their lives (and accordingly, their daily social interactions), and they rarely get to talk about themselves and how they are feeling [155]. These findings provide important evidence for informal caregivers (and emotionally distressed people in general) behaviour during prolonged interactions with social robots, and how it could potentially reduce suppressive regulatory behaviours when being engaged in self-disclosure to a social robot over time.

Due to the size, complexity and richness of the data collected as part of this study (almost 1000 data units of rich disclosures), performing in-depth qualitative assessments of these disclosures is beyond the scope of the current research. We acknowledge the need to analyse the content of these interactions qualitatively to have a deeper understanding of the nature of informal caregivers’ self disclosures’ to social robot, what sort of information these disclosures consist of (i.e., addressing explicitly the caregiving experience vs. addressing other events in their lives). This will allow us to determine in a more causal fashion whether these sort of interactions support informal caregivers to avoid suppression via self disclosing to a social robot. Moreover, addressing these questions using qualitative methods will provide us with additional tools to describe self disclosure beyond quantities and subjectively reported perceptions. We aim to follow up this systematic work with a qualitative analysis for a sample of the interactions, while treating participants self-disclosures in a careful, ethically responsible way [see [156]].

### Social Robots for Interpersonal Emotion Regulation

Beyond avoiding suppression, the results here highlight the potential and effectiveness of self-disclosing to a social robot as a constructive form of interpersonal emotion regulation. As participants were self-disclosing increasingly more to Pepper over time, they also reported that their mood positively changed after their interactions, finding Pepper to be more comforting, and feeling less lonely and stressed over time. In terms of cognitive emotion regulation and cognitive change, informal caregivers reported being more accepting of their caregiving situation, reappraising it more positively, and experiencing lower feelings of blame towards others. These findings provide further valuable evidence for the positive outcomes of employing a social robot as an intervention supporting people’s well-being and coping with emotional distress. Our results here add to previous studies [e.g., [98, 103–105, 110, 112, 113]] that show the benefits of using robots for emotional support. In addition, these findings contribute to our general understanding of how acts of communication towards a social robot, like self-disclosure and social sharing can improve people’s emotional well-being. These findings contribute to the introduction of social robots as conversational partners, and how this type of verbal interaction could support people with emotion regulation by talking about stressors and well-being. Simple tasks, like the one described in the study, are relatively easy to administer automatically in HRIs (by focusing on providing general and broad responses to users’ disclosures) but can simulate effective procedures via self-disclosure. Accordingly, social robots can offer meaningful opportunities for self-managed interventions designed to support people’s emotional health and well-being.

Our results further help to identify specific cognitive emotion regulation strategies that may be impacted by self-disclosure interactions with social robots. By showing that the cognitive emotion regulation strategies of increased acceptance, positive reappraisal and reduced other-blame were all positively impacted by self-disclosure to a social robot, we have identified specific strategies that may be impacted by this type of intervention. Accordingly, our results here suggest potential applications for social robots in real-world settings, providing crucial evidence and laying foundations for future interpersonal emotion regulation interventions with social robots to take place with informal caregivers and other emotionally distressed individuals.

Reflecting on the map of interpersonal regulation [15], our findings provide substantial evidence for the benefits and effectiveness of self-disclosing to a social robot as an intrinsic regulatory process that can be response-dependent or independent. One assumption is that despite Pepper’s limited responses, participants found Pepper to be more comforting, and might have been engaged in self-disclosing to Pepper as a form of intrinsic response-dependent regulation. Together with the positive influence the interactions had on participants’ emotional well-being and cognitive emotion regulation, we can assume that Pepper’s empathic responses supported participants coping efforts to a certain extent. Another assumption can be that due to Pepper’s limited responses, participants used the intervention as a platform for intrinsic response-independent regulation. Participants used their interaction with Pepper as a convenient space to share their emotions regardless of Pepper’s responses, engaging in a regulatory behaviour that is similar to affect labelling [59] and other emotional introspective processes with users self-reflecting on their emotions and behaviours [61]. These results signify the potential of deploying social robots as listeners and highlight how people respond and behave to these embodied artificial agents when in need to be heard [see [57, 58]]. This is especially meaningful in the context of informal caregiving as informal caregivers rarely have the opportunity to express their feelings or talk about themselves, while their care recipient is often in the spotlight [155]. Here we showed how a relatively simple intervention with a widely available social robot could provide a convenient channel to talk about their own emotions feelings, sharing about themselves and for once not about the care recipient.

It is important to mention that in order to further understand the regulatory mechanisms that underpin any positive impacts brought about by this intervention, we must further inspect the qualitative data we collected, open-ended answers from the participants and our own observations. Furthermore, future researchers are encouraged to further inspect these two regulatory mechanisms in interventions with social robots using experimental procedures, casually explaining whether people use their self-disclosures towards social robots for response-dependent or independent intrinsic regulation.

### Relational Engagement with Social Robots Extends Familiarity

While it might be intuitive that self-disclosure would increase over time in traditional counselling interaction [157], our study extends this understanding to HRI, an area where such outcomes are not necessarily expected or well-documented. Contrary to common novelty effects observed in many HRI studies, where engagement might diminish over time [158, 159], our results show a consistent increase in self-disclosure over multiple sessions, consistent with our previous results [1]. This pattern suggests that participants not only maintain interest but also deepen their engagement, which is indicative of building a therapeutic relationship with the robot, not merely habituating to it. Furthermore, we extend the discourse beyond mere exposure by illustrating that repeated interactions with the social robot lead to increased perceptions of the robot’s social presence and competencies, paralleling the development of rapport in human relationships. While humans can utilise a variety of intuitive and automatic social cues to encourage and nudge others to self-disclose [160], current-day robots are far more limited in their repertoire of expressions [49]. Moreover, robots are limited from using techniques practised by human counsellors to encourage patients to engage in self-disclosure [115, 161], such as practitioner self-disclosure and sharing personal insights [162, 163]. Despite these limitations, our study shows that social robots can still foster a meaningful relationship over time in intervention settings. This suggests potential for future advancements in HRI to develop more sophisticated social robots, enhancing their effectiveness in therapeutic settings. This significant finding challenges the conventional views in HRI, which often expect diminished engagement due to the novelty wearing off [158, 159]. The study’s findings contribute uniquely to the understanding of HRI by showing that increased self-disclosure does not merely stem from repeated exposure but aligns more closely with mechanisms observed in ongoing human counselling relationships. This challenges the typical usability-focused interpretations in human-computer interaction (HCI) and underscores the potential for social robots to go beyond simple task-based interactions to engage users on a relational level.

Our findings suggest that the dynamics of building a therapeutic relationship may be fundamentally similar, whether the interaction is with a human or a robot. This similarity is particularly promising for the future of social robots in therapeutic roles, indicating that humans’ response and adaptation may fulfil similar relational functions presented towards human professionals in care settings [157]. This emphasizes the potential for social robots to be perceived not merely as tools but as social entities capable of supporting therapeutic processes. Overall, the observed pattern of self-disclosure with a social robot reflects not just a familiarity effect but can be used as an objective indication for the establishment of rapport and trust over time. This highlights the potential for social robots to engage users on a relational level, supporting therapeutic processes and contributing to emotional well-being.

### The Implications of Prolonged Use of Robot-Assisted Emotional Support

In our study, we observed significant positive impacts of robot-assisted interventions on the emotional well-being of informal caregivers. Participants reported improved mood, reduced feelings of loneliness, and decreased stress levels after interacting with the robot. However, it is crucial to address potential risks associated with prolonged exposure to such technology, particularly the risk of overreliance on robots for emotional support. While the data suggest that interactions with the robot can enhance emotional regulation, there is still a valid concern that caregivers might develop an attachment to the robot and rely on it excessively [51]. This overreliance on *“others"* could detract from the use and development of essential *intrapersonal* emotion regulation strategies [164–166], such as self-reflection and mindfulness, which are critical for long-term psychological resilience [7, 167]. This concern is especially relevant for caregivers, as previous studies have highlighted their challenges in accessing digital interventions [168]. An always-available robot, designed for natural interaction, might exacerbate overdependence and attachment.

To mitigate these risks, several strategies can be implemented. Introducing social robots in public settings, rather than aiming for their introduction in personal home settings, could support the safe and responsible integration of these agents. Controlled settings under the supervision of healthcare professionals allows for regular monitoring and evaluation, ensuring that the interactions complement existing therapeutic practices rather than replace them. Additionally, the development of formal policies and operational guidelines, like the recent EU regulations on AI [see [169]], can provide a structured framework for safe and effective robot use [101]. Future policy and guidelines could include measures to ensure that robots are used as supplementary tools in a constructive manner. Moreover, educational programs for caregivers can also play a crucial role in emphasizing the importance of balancing technology use with traditional emotion regulation strategies [92, 170]. By fostering a more holistic approach to managing emotional distress, these programs can help caregivers utilize the benefits of robot-assisted interventions without becoming overly dependent on them.

Future research should explore the negative implications of prolonged robot interactions, focusing on attachment tendencies and comparing the efficacy of robot-assisted interventions with traditional therapeutic approaches. Such studies will help defining the boundaries within which robot interactions remain beneficial and identify when they might start hindering the development of personal emotion regulation skills. By incorporating these strategies and continuing to explore the complexities of HRI, we can maximize the benefits of robot-assisted interventions while ensuring that they support, rather than hinder, the development of essential human skills.

## Limitations and Future Research

### Novelty Effects and Sustained Engagement

Our study duration extended over five weeks with ten sessions which is considered relatively long and intense for HRI experiments [101], allowing us to uniquely explore sustained engagement. Typically, HRI assumes a ’*honeymoon period*’ [see [158]] in initial interactions, where heightened engagement is driven by the novelty of interacting with robots. This excitement is expected to wane as familiarity increases. Nevertheless, it is important to address a critical limitation arising from the unclear definition and duration of novelty (i.e., the ’honeymoon period’) within HRI research. While our results suggest that participants may have moved past the initial novelty effect, it remains possible that we are still observing behaviours within an extended novelty phase. The five-week study period, and ten sessions in total, might not have been long enough to detect a decline in the initial fascination that comes with interacting with a robot for the first time. The results observed in this study could also be attributed to the heightened attention participants allocated due to the novelty experienced compared to their everyday life experiences.

In HRI, where the task is of a social nature rather than mere usability as seen in HCI [40], we can often expect to observe signs and trends indicating that novelty wears off earlier during prolonged interactions, due to the influence of social norms and expectations shaped by previous interactions with humans [171, 172]. When interacting socially, people tend to quickly form impressions and follow social behaviours, often making judgments within just a few encounters or even moments [173]. While interactions with robots are still conceptually distinct from those with humans [46, 174], and novelty is not impression management [159], similar cognitive mechanisms are involved [45, 46, 174–176]. Therefore, we assume that the effects of novelty will resolve at a relatively early stage of prolonged interaction when the task is inherently social. Indeed, previous research has highlighted similar trends in long-term interactions with other artificial agents that interact socially with humans, such as chatbots, which respond to different embodiments [e.g. [136, 177, 178]]. However, our findings challenge this assumption, demonstrating sustained and even increasing levels of engagement. These results suggest that the interactions provided substantive value beyond the novelty, also serving as effective and meaningful interventions. Since we observed a gradual increase in participants’ social perceptions of Pepper over time, we assume that if it were merely a matter of attention, the novelty effect would have occurred more immediately rather than gradually. Nonetheless, our results are open to interpretation and should be further validated, as the scope and definition of novelty in HRI remain unclear.

This ambiguity underscores a significant gap in the field, as there is not enough evidence from long-term HRI deployment to fully capture if engagement is due to novelty or effective deployment, or whether the timeframe of deployment or number of interactions is within the novelty phase. Accordingly, our field lacks a standardised metric or benchmark to conclusively determine when the novelty effect diminishes, and how to identify it. This is especially noticeable when considering that long-term studies with disembodied agents can overcome some logistical limitations of long-term deployments of social robots. For example, Skjuve et al. [177, 178] studied long-term interactions with Replica for 12 weeks, which is considered long among HRI studies [101, 179]. Therefore, the results of our study set a precedent for future investigations to extend the duration and perhaps increase the frequency of interactions to better understand the dynamics of long-term human-robot relationships. In spite of that, it would also require to follow careful ethical consideration. Would additional sessions continue to provide value, or would they reach a point of diminishing returns? This is a significant consideration in the design of future studies and in the broader application of robot-assisted interventions. Hence, in addition to advocating for more long-term HRI deployments in research, we should also prioritize comprehensive and up-to-date reviews and meta-analyses of these deployments and their effects. This approach will help researchers in the field interpret their results and determine whether their findings stem from novelty or effective deployment.

While considering the appropriate dimensions of novelty in HRI, and when novelty wears off, it is important to note that our intervention here was intentionally designed as a fixed set of sessions, following the conventional practices for studying technology-based psychosocial interventions [180–182]. These are often tailored to optimise engagement and well-being outcomes within practical constraints [183]. In the context of interventions with social robots, this approach is particularly applicable given the current limitations in robot autonomy and social capabilities. Social robots are programmed for specific, structured interactions which do not yet support indefinite, autonomous engagement without human oversight [101]. Thus, beyond understanding novelty within the field of HRI, researchers should consider how novelty perceptions and effects play out within the specific, structured interactions they deploy in their own research. These insights could inform methodological choices and reflect on the current state of technology in social robotics. This is especially important when developing interventions, as these have clear goals and endpoints and should follow established protocols based on evidence suggesting the optimal duration for efficacy [180, 181, 184]. This approach helps in managing resources efficiently and setting realistic goals and expectations for participants and practitioners. Addressing these considerations is crucial as we plan future studies, aiming to explore the potential for extended interactions and optimally structure interventions to balance engagement, efficacy, and the practical capabilities of current social robotic technologies. By expanding the scope and scale of HRI studies, we can better understand and redefine the boundaries of novelty and engagement in human-robot relationships, ensuring that future social robotic interventions are both effective and meaningful over prolonged periods.

### Mediated Embodiment and Experimental Control

Our study involved interactions with Pepper via video mediation rather than face-to-face, which might influence perceptions and behaviours differently [185]. We chose online-mediated interactions to allow access for a limited demographic, such as informal caregivers, who may not easily access in-person sessions due to their care responsibilities [81, 186, 187]. When conducting this study, we envisioned how social robots could serve as viable support in both public and intimate settings, where a session or two a week could offer a practical and effective solution in physical spaces. As social robots are increasingly integrated into care settings to support care *recipients* and are envisioned as critical agents in these environments [102, 188–190], our study aimed to explore how these robots could also be utilised to support care*givers*. Therefore, despite virtual agents being cheaper to implement, we opted for a social robotic intervention to provide evidence that will support future social robot deployments in these settings and for such interventions.

Recent experimental studies have shown minimal differences in participants’ perception and behaviour between in-person and mediated interactions with social robots [e.g., [50, 191–197]]. We assume that this trend is further reinforced by the widespread adoption of computer-mediated means of communication during the Covid-19 pandemic, which made online interactions more commonplace [198] and, therefore, made our experiment more reflective of the prevailing social context. Despite the lack of physical co-location, the cues of physicality demonstrated by Pepper, even when mediated, provides a richer medium than virtual agents [see [50]], enhancing user engagement through three-dimensionality and tangible mediated presence [93, 199]. For informal caregivers, due to persistent burden, the less demanding nature of natural interactions with a physically present robot, even in a mediated form, can significantly improve perceived support and engagement levels [30].

Furthermore, the concept of perceptual authenticity plays a critical role in how users engage with social robots like Pepper. The physical embodiment of Pepper, even when mediated through video, enhances cognitive and social engagement by providing tangible cues that virtual agents cannot replicate. This aligns with Media Richness Theory [93], which posits that communication media differ in their ability to effectively convey information; a physically embodied robot offers a richer medium, fostering more naturalistic human responses. This is particularly important in therapeutic contexts, where the realism and immediacy of interactions significantly influence the effectiveness of the intervention. As we see a surge in the deployment of LLMs in a variety of embodied [e.g., [150]] and disembodied agents [e.g., [200]], more research is needed to verify the cognitive differences in processing and reacting to information provided by agents of different embodiments. We encourage researchers to replicate our study with agents in different embodiments (e.g., avatars, chatbots, physically present robots) and to further reflect on how these results might change accordingly.

Another limitation that should be discussed in our study, is the lack of control condition, which could have strengthened comparisons against other forms of interaction [201]. Since this study aimed to replicate our previous investigation, we recognized that the between-subjects design used in the earlier study did not yield the desired results. In planning this replication with informal caregivers, we recognized the significant challenges in recruiting this population due to their care-related responsibilities [202–204]. Moreover, our primary objective was to investigate the interaction dynamics and potential of live, dynamic exchanges with a social robot, specifically focusing on the nuanced responses that a humanoid robot like Pepper can elicit. Implementing a control condition that accurately mimics or parallels the complex, nuanced interactions with a social robot-without introducing confounding variables-posed significant challenges. Instead, we employed a within-subjects design, where each participant’s experience served as their own control. This approach allowed us to monitor changes over time, focusing on how repeated interactions with Pepper influenced perceptions and behaviours, and well-being overtime [205, 206]. This decision ensured a robust assessment of the intervention’s value and effectiveness, while maintaining methodological robustness and experimental power [207]. Future research is encouraged to further validate the behavioural paradigm introduced here and in [1] by testing agents with different embodiments or social robots that respond to varying behaviours. This would allow for comparative analyses and further validation of the findings presented in this study. In addition, the results of the study can be compared with existing and future literature that employs various speech-based interventions and interactions over time with other agents, such as humans or disembodied agents [e.g., [136]].

### Mitigating Confounding Factors in Diverse Caregiver Samples

Our study included a diverse sample of informal caregivers, with an age range of 19 to 63 years. This broad age spectrum introduces variability in life experiences that may influence caregivers’ experiences. While this diversity enhances the generalizability of our findings across the informal caregiver population, it also presents specific challenges. Notably, the differing life stages of caregivers could be assumed to influence how they perceive and manage emotional distress [e.g., [208]]. Conducting behavioural experiments within specific populations, such as informal caregivers, comes with inherent complexities [202–204]. The heterogeneity of this population in terms of demographics and caregiving situations necessitates some level of compromise in sample homogeneity.

Addressing these challenges, our experimental design utilizes a within-subjects approach where each participant serves as their own control. This design significantly reduces the potential for confounding factors, as each caregiver’s response is compared against their own baseline, rather than across different individuals [205, 206]. This method helps us isolate the effects of the intervention by controlling for individual differences that exist prior to and during the intervention [209]. Further addressing variability within our sample, we implemented mixed-effects models in our statistical analysis. These models are particularly effective in longitudinal studies like ours where repeated measures are taken from the same subjects over time. By accounting for random effects, we control for individual differences at the subject level that could influence the outcome measures (e.g., differences in age, socio-economic status, identified gender, etc.), thus providing a robust analysis that accounts for the nested structure of our data and offering a more accurate representation of the intervention’s impact [210, 211]. In other words, mixed-effects models enable us to account for both fixed effects, which are consistent across subjects, and random effects, which vary between subjects, thereby improving the accuracy and generalizability of our findings [210]. This dual-level modelling approach is particularly useful for our study as we are interested in identifying individual differences due to the interventions. By effectively modelling these individual variations, we can better understand how different subjects respond to the intervention [211]. Finally, the core of our research centres on the shared aspects of the informal caregiving experience-specifically, the emotional distress and coping mechanisms employed by caregivers. These common experiences, despite varied personal backgrounds, highlight the relevance of our findings within this wider demographic.

Our experimental design and statistical approach establish a strong basis for internal validity, which refers to the degree to which the results of the study are attributable to the intervention rather than to other confounding variables. By rigorously controlling for individual differences and employing robust statistical techniques, we increase confidence that the observed effects are genuinely due to the intervention. This also supports generalising findings within certain broad parameters of informal caregivers. At the same time, the external validity-or more specifically, the generalisability across specific subgroups of informal caregivers, such as younger caregivers or those caring for individuals with specific conditions (e.g., dementia)-remains nuanced due to the heterogeneity in caregiving contexts. To further this research, future studies could replicate this study and explore the results within specific subgroups, such as younger caregivers or those caring for individuals with specific conditions (e.g., dementia or old age). By conducting targeted replications or secondary analyses on the data collected in this study, we can move from inductive findings towards a more deductive approach that tests these findings across different contexts. This dual strategy would enable more precise tailoring of interventions to meet the diverse needs of caregivers from different populations more effectively.

### Challenges in Measuring Self-Disclosure

In this study, we recorded and transcribed interactions to quantitatively measure the volume of disclosures, capturing both duration in seconds and word count. This decision was driven by the challenges associated with manually coding and analyzing a substantial dataset comprising 993 data units, each representing an instance of interaction between participants and the social robot. Previous research supports our approach, suggesting that the volume of disclosure can serve as an indicator of the depth and quality of self-disclosures [153, 154, 212]. Despite this, the limitations of not capturing the emotional depth and content specificity of the disclosures should be acknowledged.

Studying self-disclosure presents inherent limitations and challenges. Self-disclosure is a multifaceted construct involving both the quantity and quality of information shared, as well as the emotional and contextual nuances of each interaction [160, 213]. Quantitative measures, while useful for capturing the extent of disclosures, might fall short in conveying the richness and complexity of the content [213]. However, other methods that could help make sense of the disclosed content (e.g., quantitative and qualitative manual content analysis) can be labor-intensive and subjective, potentially introducing biases [214]. Balancing the need for comprehensive analysis with the constraints of objectivity and feasibility remains a significant challenge in this area of research [160].

The complexities involved in a detailed content analysis of the disclosures [see review, [160]] led us to prioritize automated measurements of disclosure volumes to ensure objectivity and manageability. This approach minimized potential subjective biases and enhanced the reliability and consistency of our data analysis. Since our main objective in this study is to assess the behavioural outcomes of the intervention, this method enabled us to systematically measure changes in self-disclosure behaviour over time and provide evidence of caregivers’ self-disclosure behaviour during the intervention. While the duration and length (i.e., word count) of disclosures provide valuable insights into the extent of engagement, they do not fully reflect the quality or depth of disclosures. To complement these quantitative measures, we included analyses related to participants’ subjective perceptions of their disclosures towards the robot (see Sect. 4.1.1). This measure offers a more subjective perspective, providing insights into how participants themselves perceived the depth and relevance of their disclosures, and indirectly reflecting the quality of disclosure [see Sect. 3.6.3, and [127]]. The results of this analysis showed that participants’ subjective perceptions of their self-disclosures increased over time (see Sect. 4.1.1 and Fig. 3), complementing the volume measures by providing a deeper understanding of the engagement’s perceived quality.

Looking ahead, we plan to conduct more direct analyses of the content and quality of disclosures in a dedicated manuscript. This will include using qualitative analyses of the content to assess the sentiment and emotional nuances of the interactions. While the current study focused on systematic behavioural measures for feasibility and objectivity, a follow-up study will examine the depth and breadth of disclosures to Pepper, offering meaningful insights into the content people tend to share with robots, not just how and to what extent they share.

## Conclusions

These findings pave the way for exploring social robots not only as conversational partners in social contexts but also as potential interventions for supporting people to cope with emotional distress. The study offers vital support for how human–robot relationships could act as ideal settings for robotic interventions for well-being. Through a long-term experiment with a target population experiencing high distress and burden [informal caregivers; [20, 23]], our study provides crucial evidence for the benefits and effectiveness of self-disclosing to social robots as means for intrinsic interpersonal regulation. informal caregivers self-disclose increasingly more to a social robot over time, perceived the robot as more social and competent over time, experienced positive mood change, felt less stressed and lonely, and cope better with the caregiving experience via adapting cognitive emotion regulation skills of acceptance, positive reappraisal, and experiencing lower feelings of blame towards others. The Human–Computer Interaction (HCI) and HRI research fields devote considerable research and development resources to eHealth solutions and health technologies in general for care *recipients*, while scant resources are allocated to eHealth solutions and digital interventions for informal care *givers* [92]. Even though they may not have a diagnosed condition, many informal caregivers are dealing with very challenging circumstances in their lives and could benefit from the assistance and support of various digital solutions. Instead of just utilizing social robots as companions [e.g., [52]], our goal was to give informal caregivers a self-managed intervention that allowed them to self-reflect on their lives and the caregiving experience via social interactions and promote social-emotional communication with a robotic emphatic agent. This aligns with the growing recognition of the importance of including informal caregivers as key stakeholders in social robotics research [215]. Finally, our study reported here further validates previous results [see [1]], extends this behavioural paradigm as a potential intervention, and adds up to previous research showing how social robots can emotionally support people in need.

## Declarations

### Availability of data and materials

Consistent with recent proposals [117, 118], we pre-registered the study and report for how we determined our sample size, all data exclusions, all manipulations and all measures in the study [see [119]]. In addition, following open science initiatives [e.g., [120]], the de-identified data set, stimuli and analysis code associated with this study are freely available online [121].
